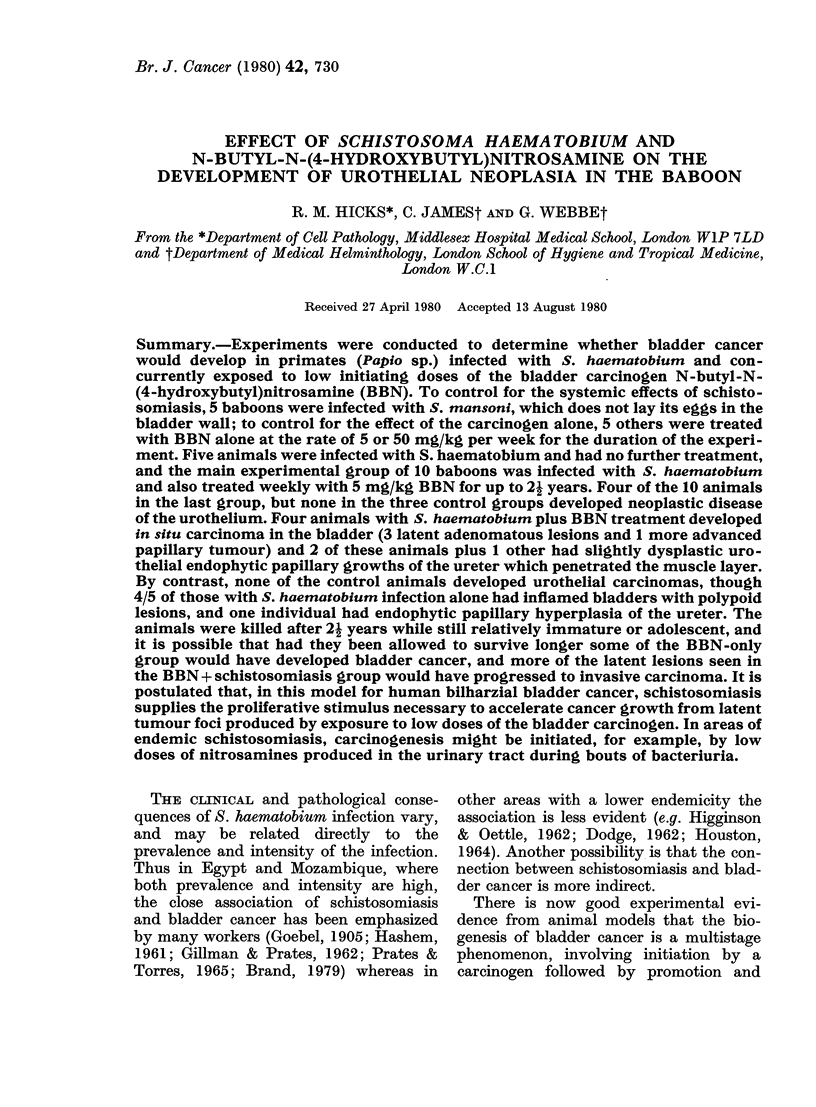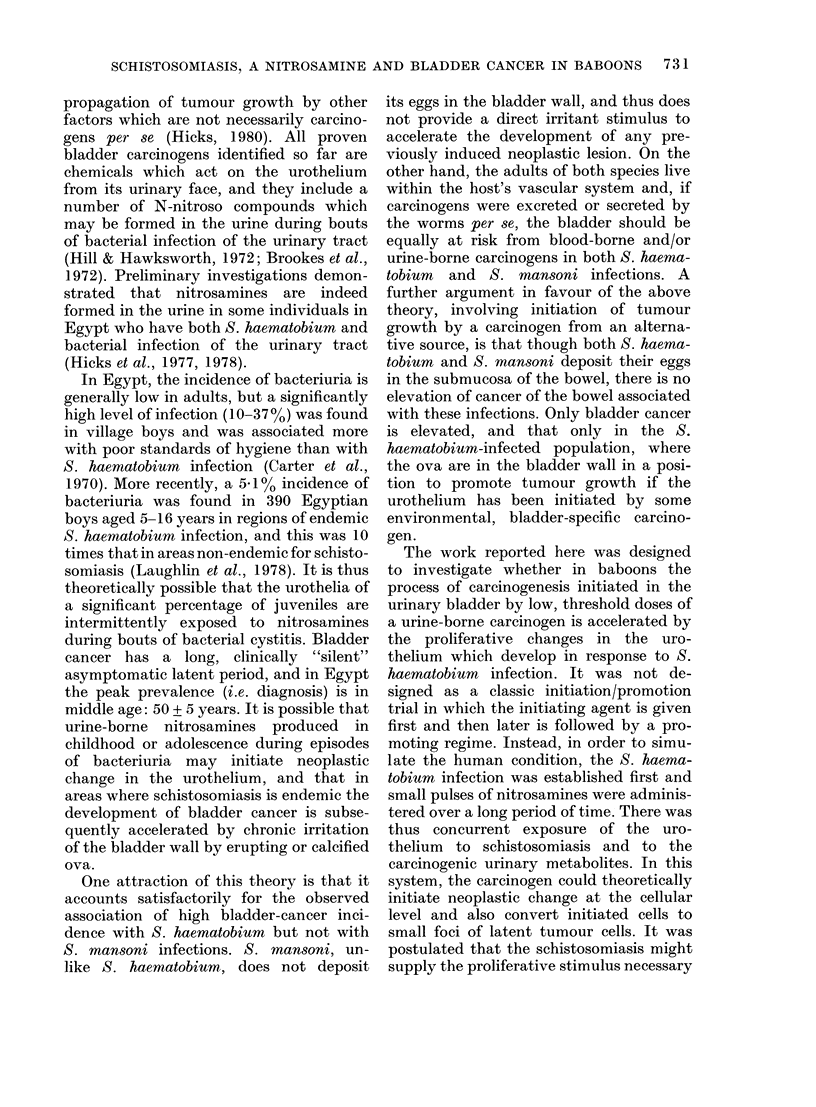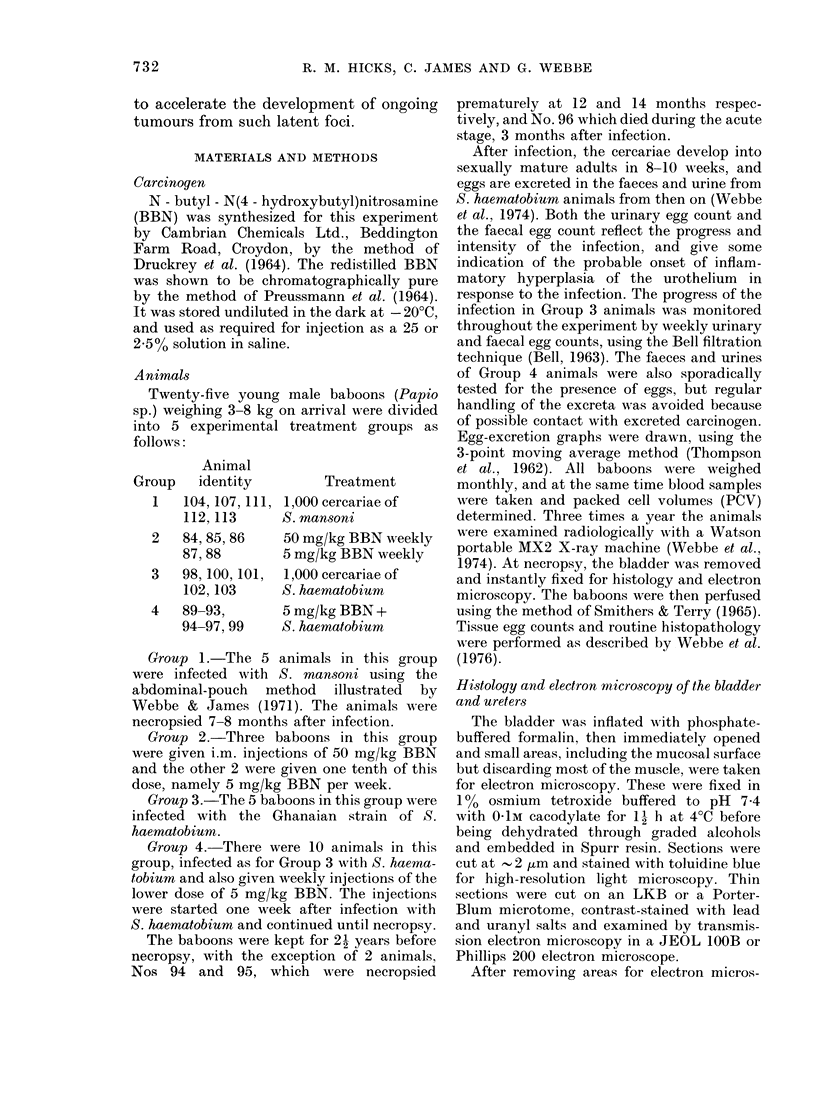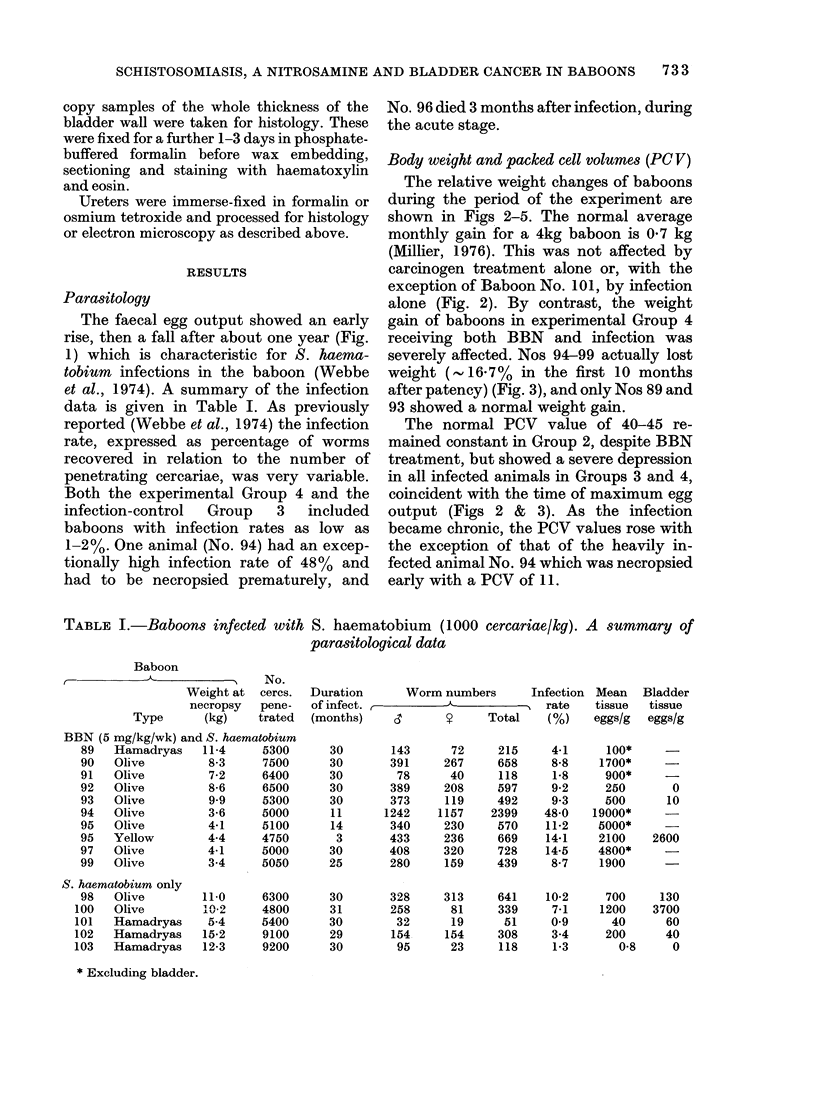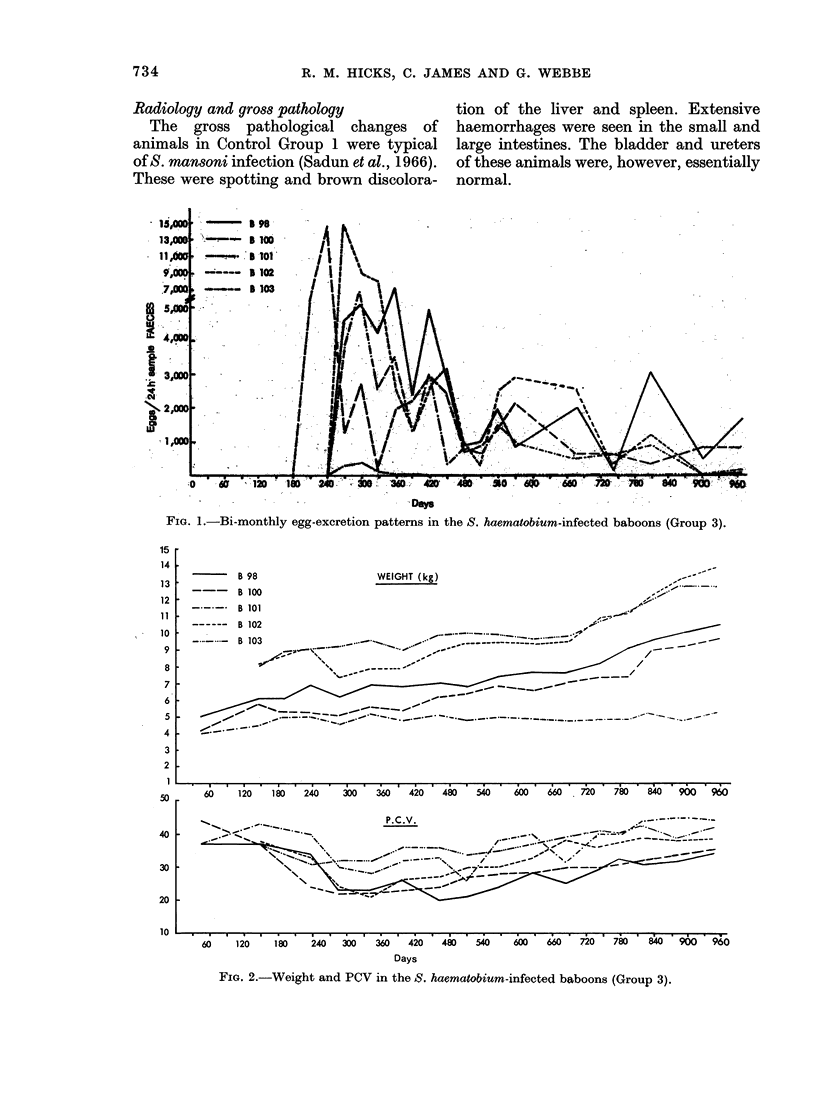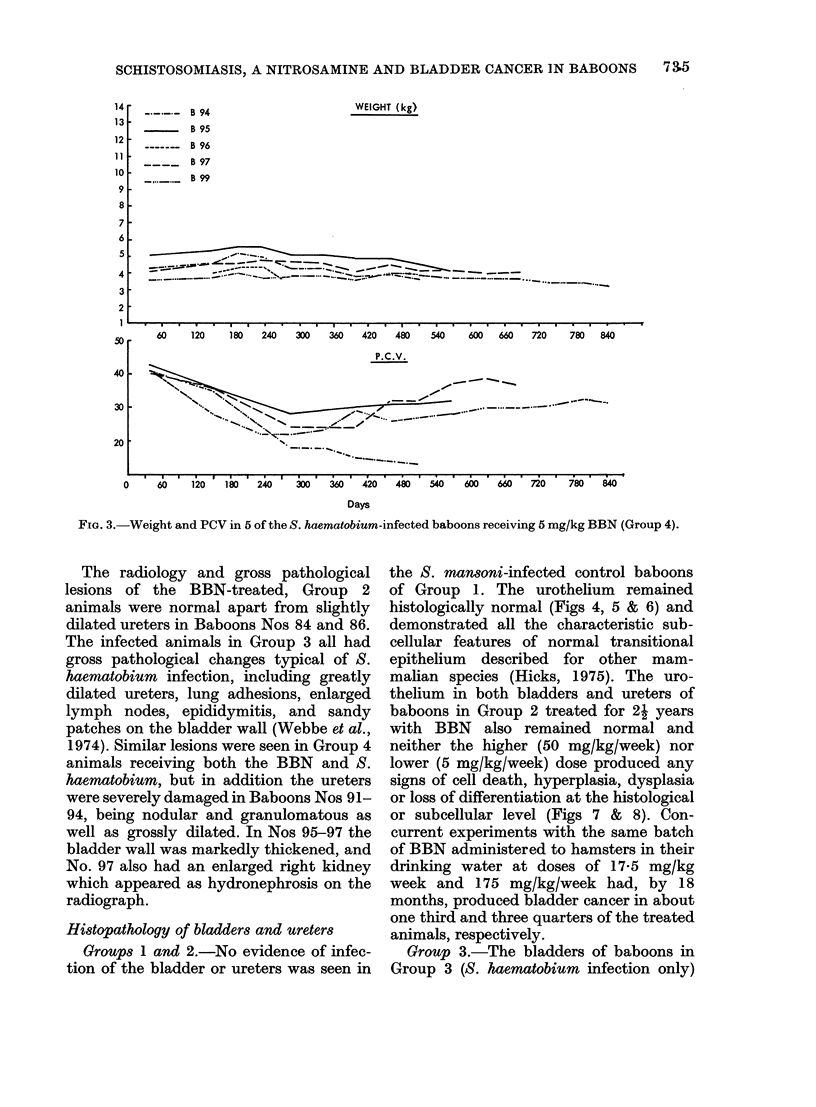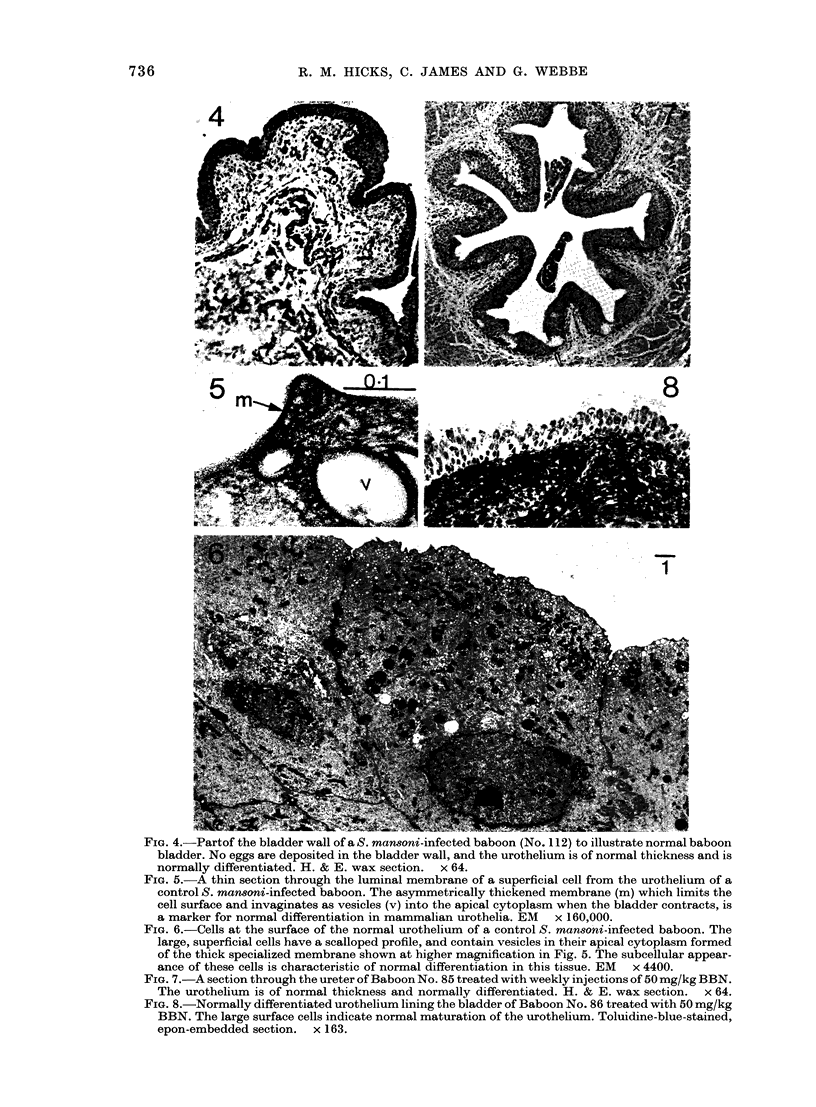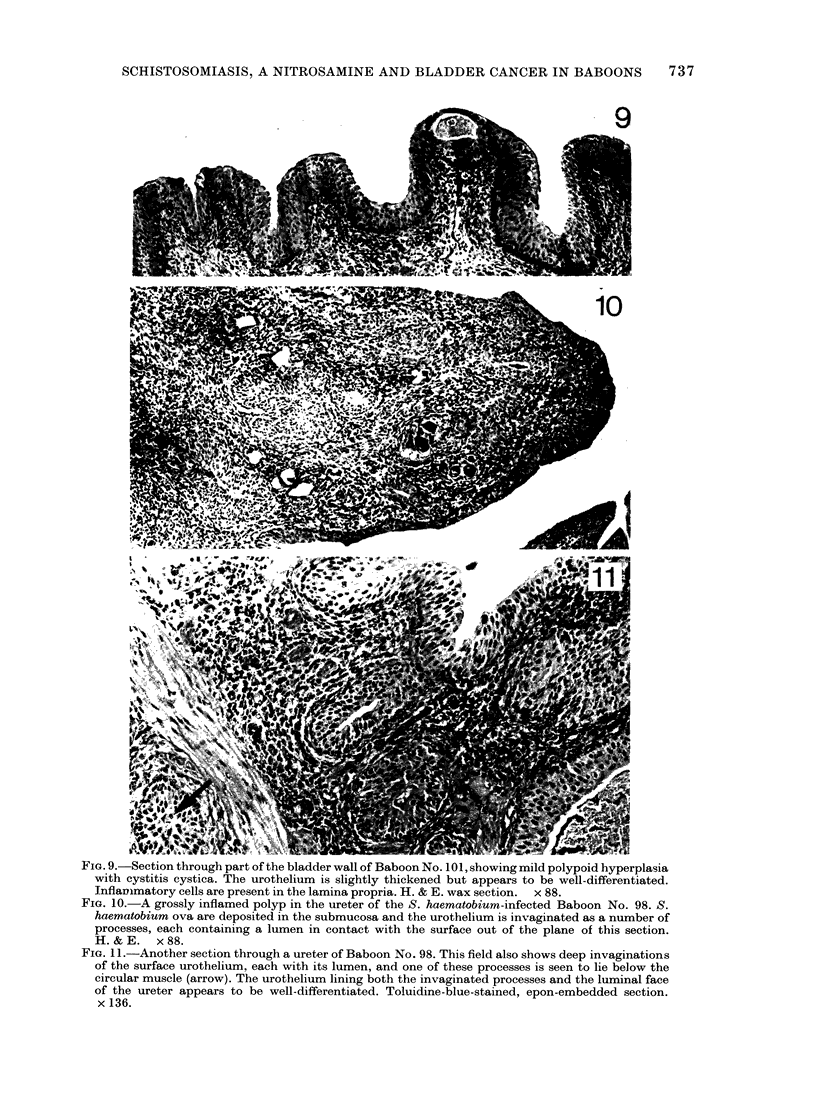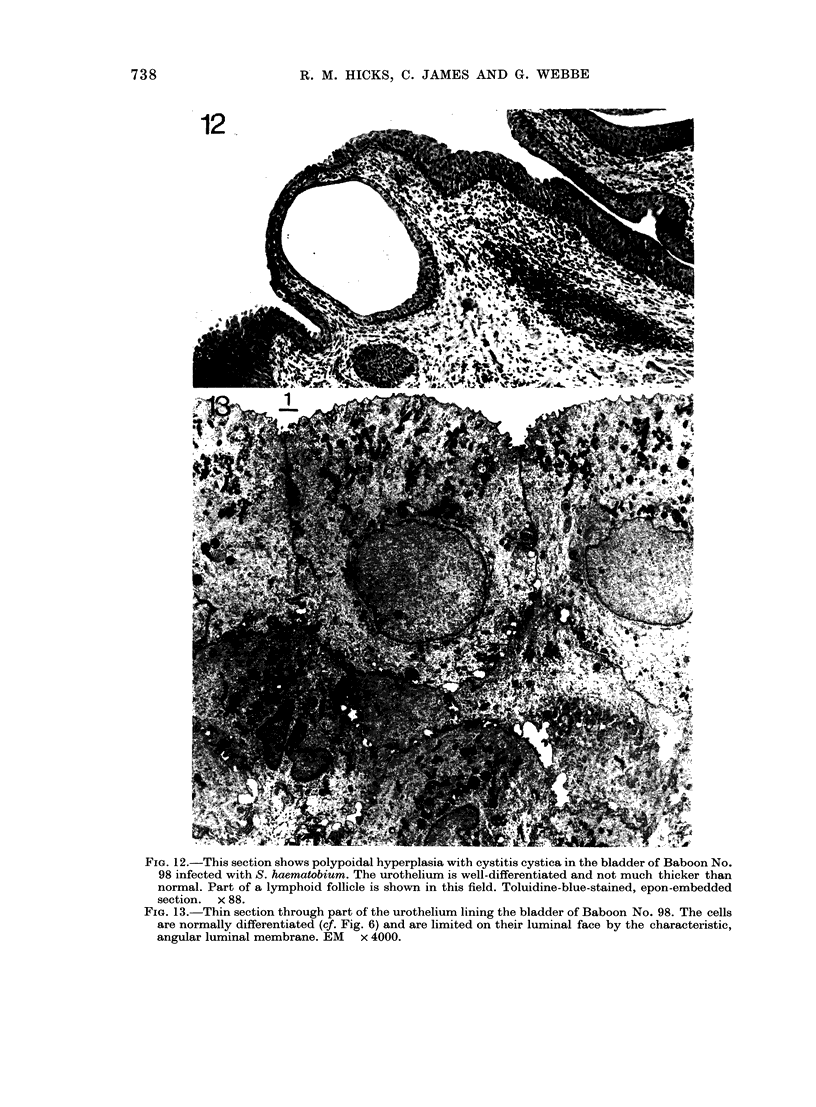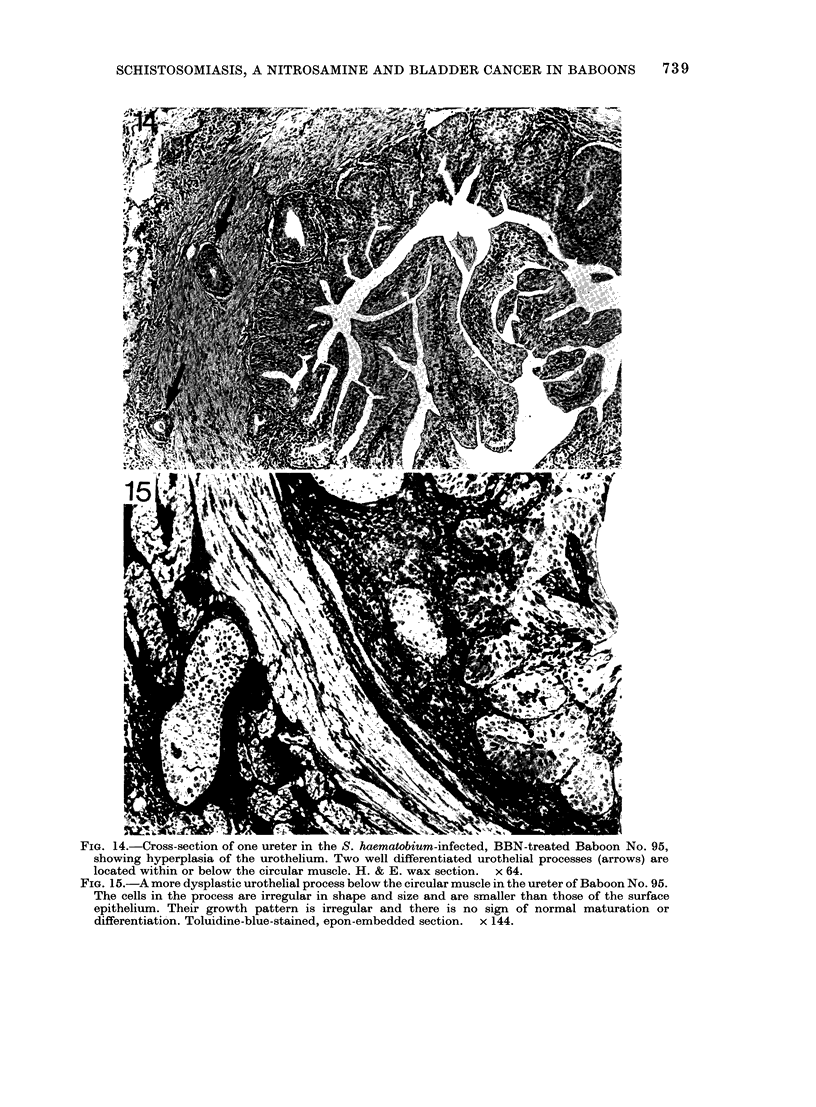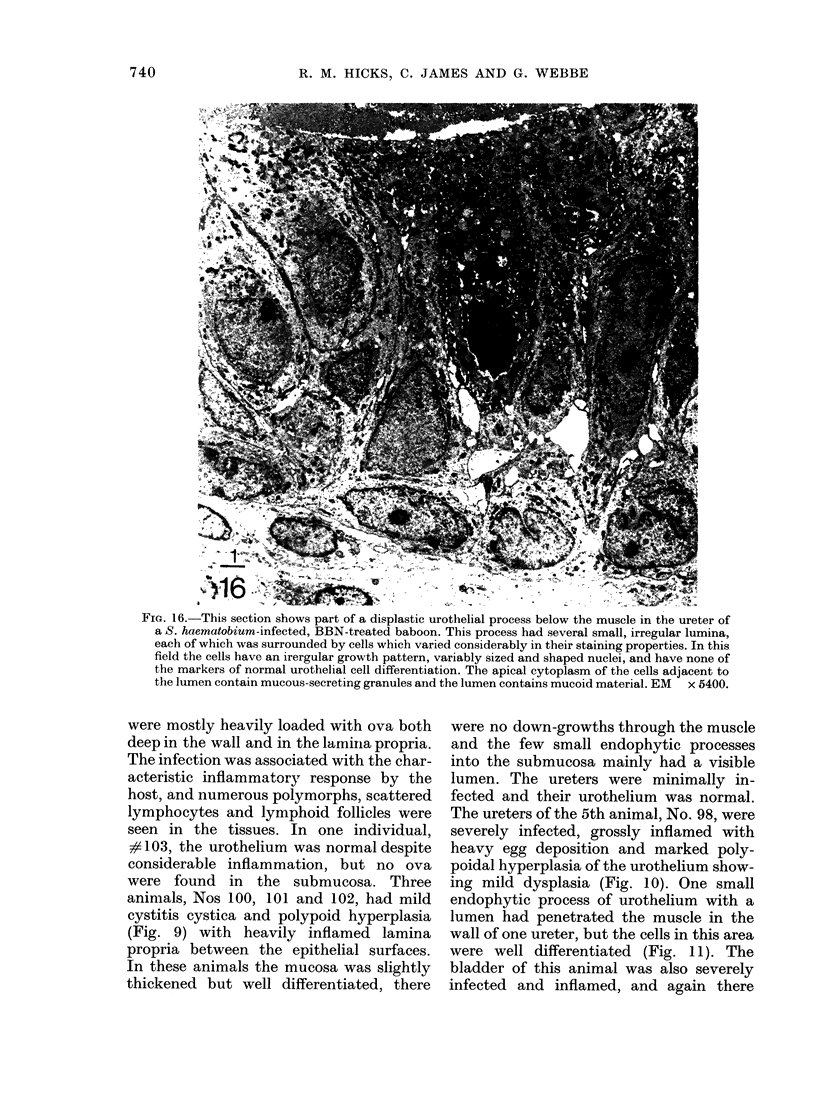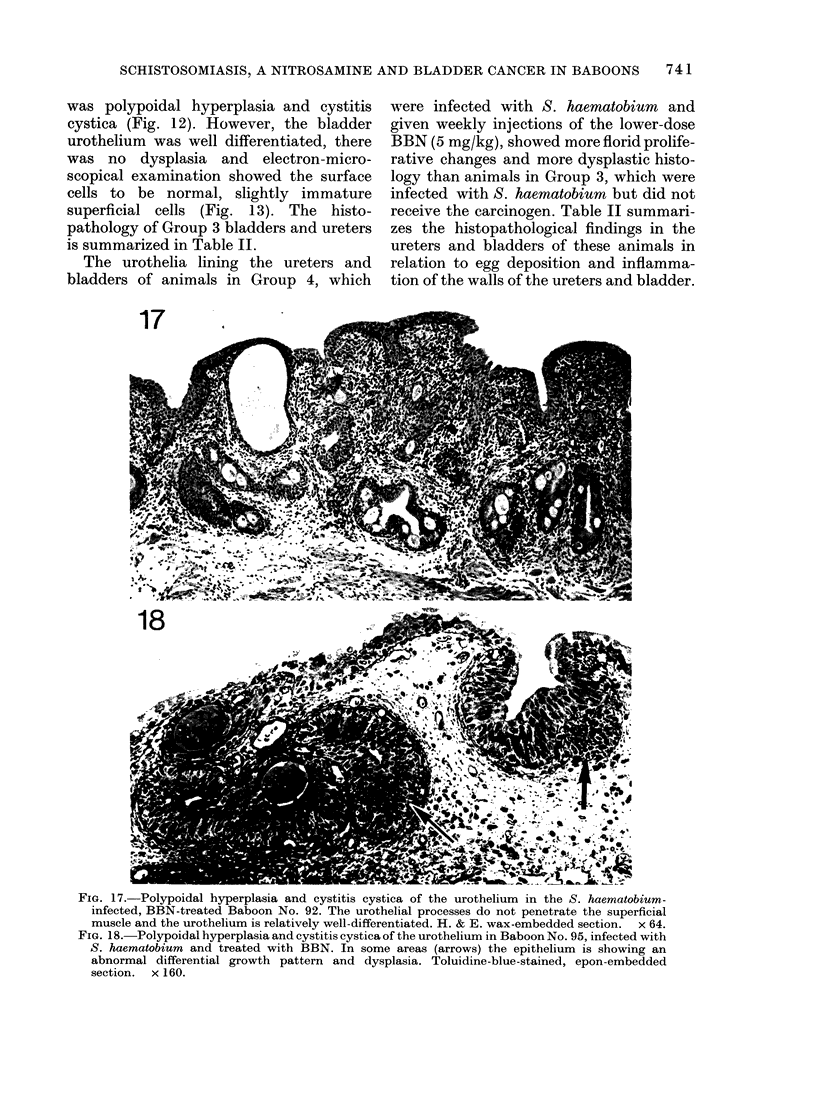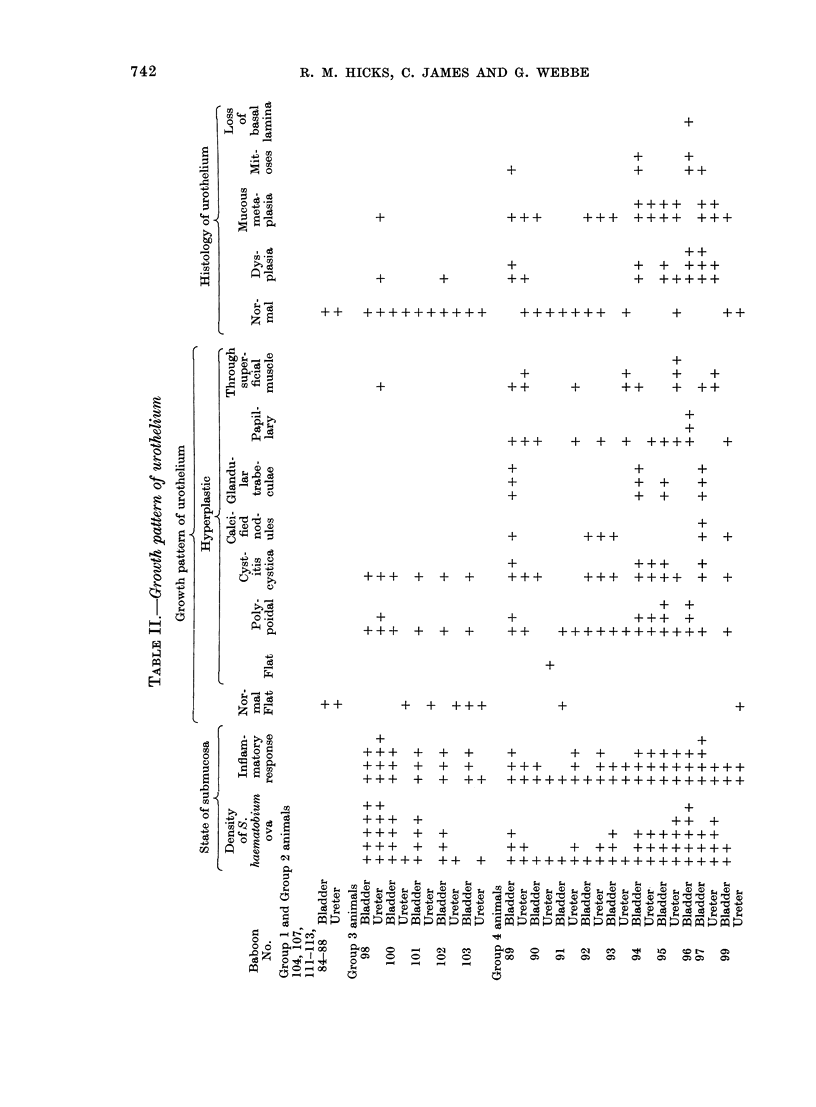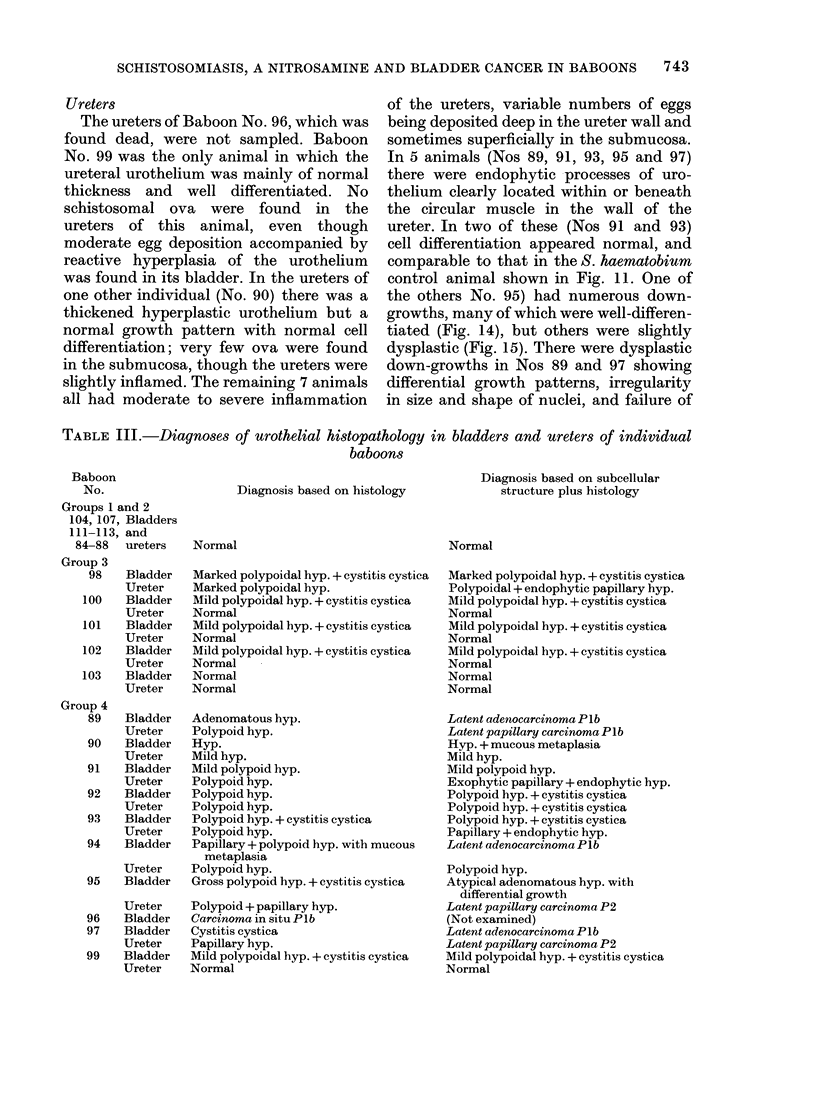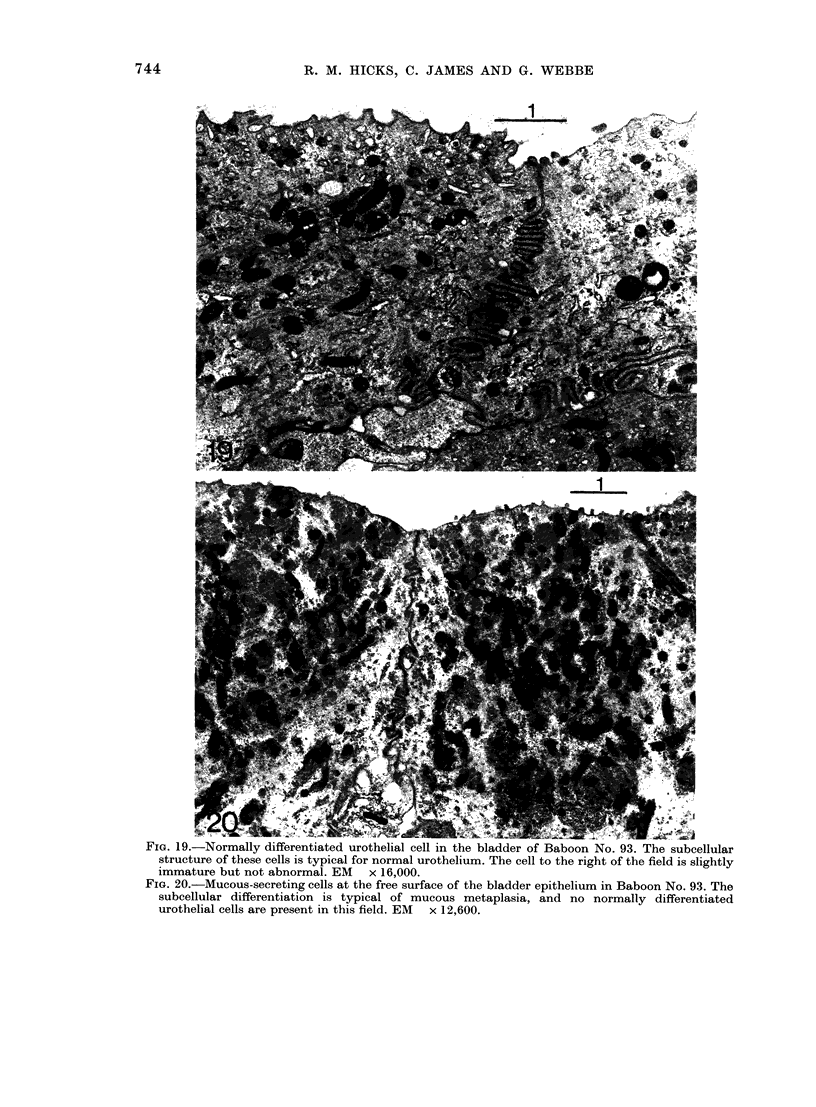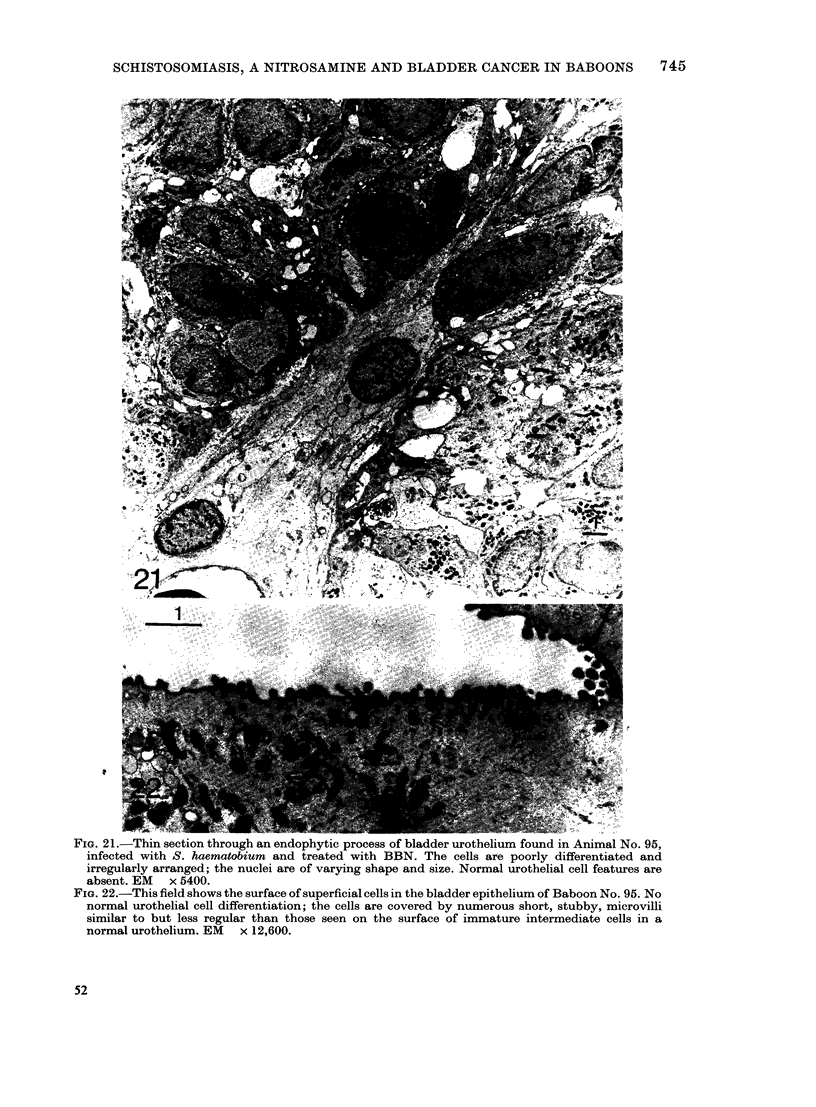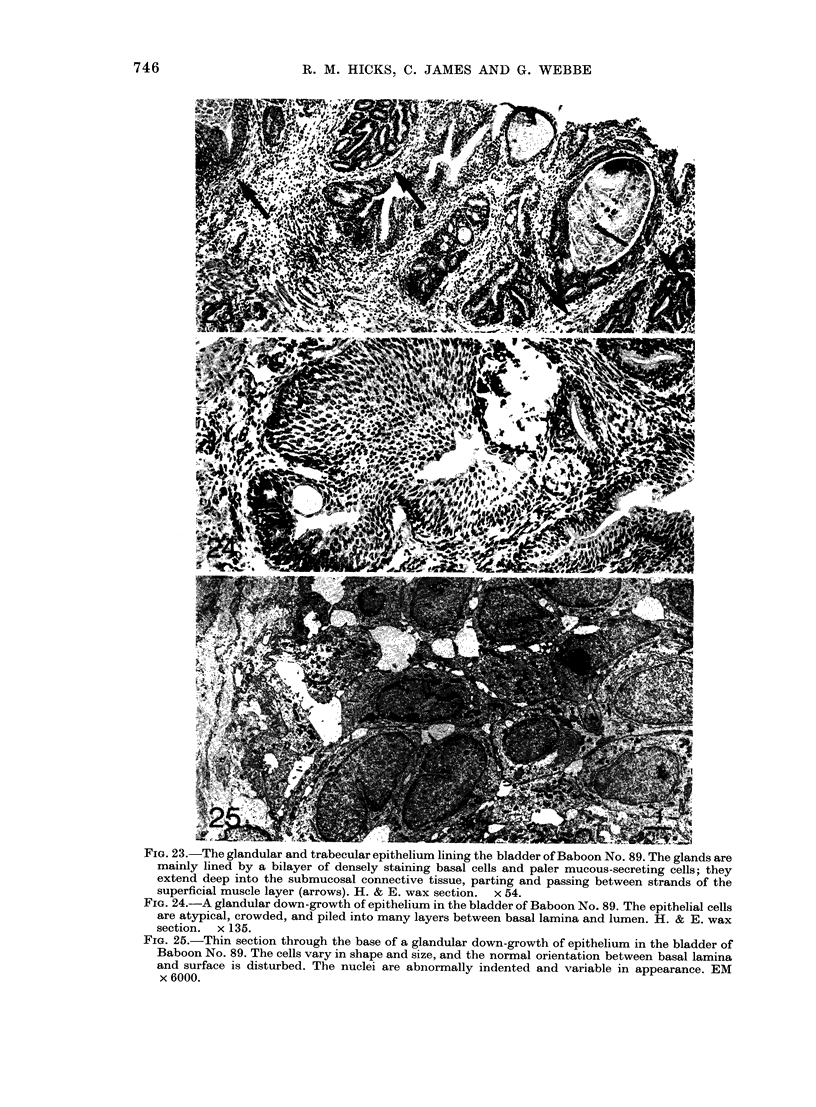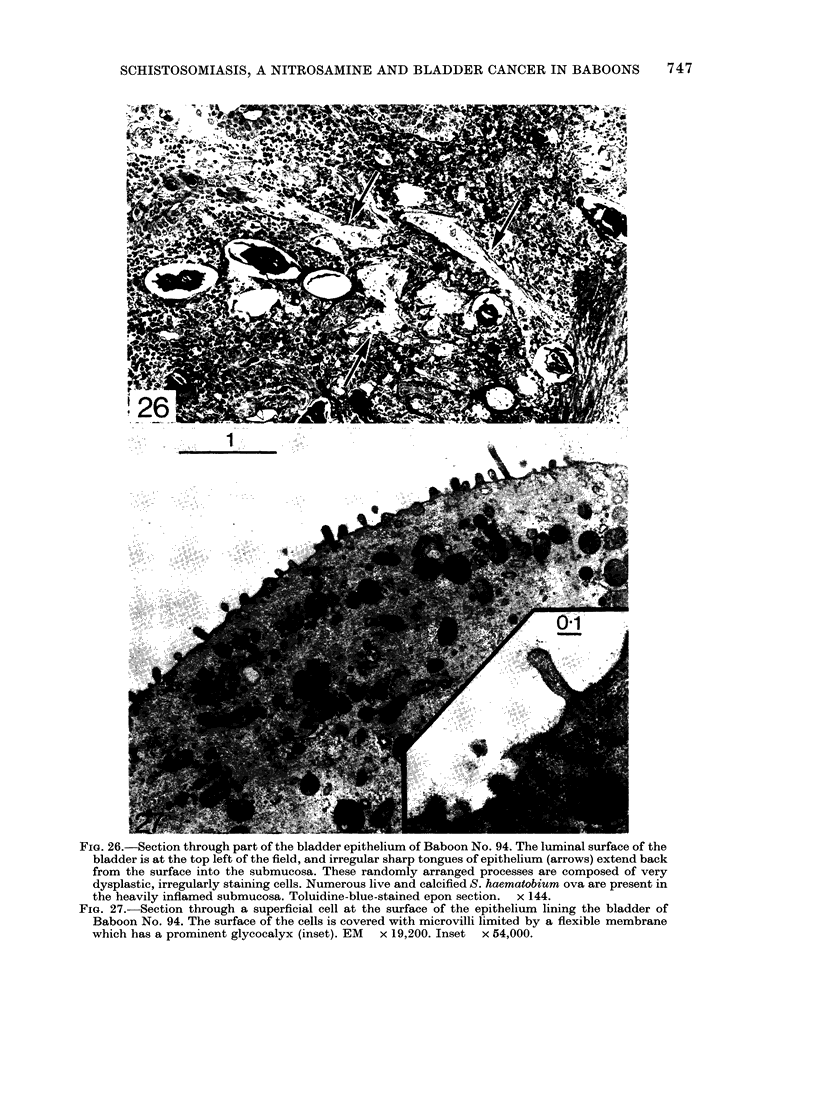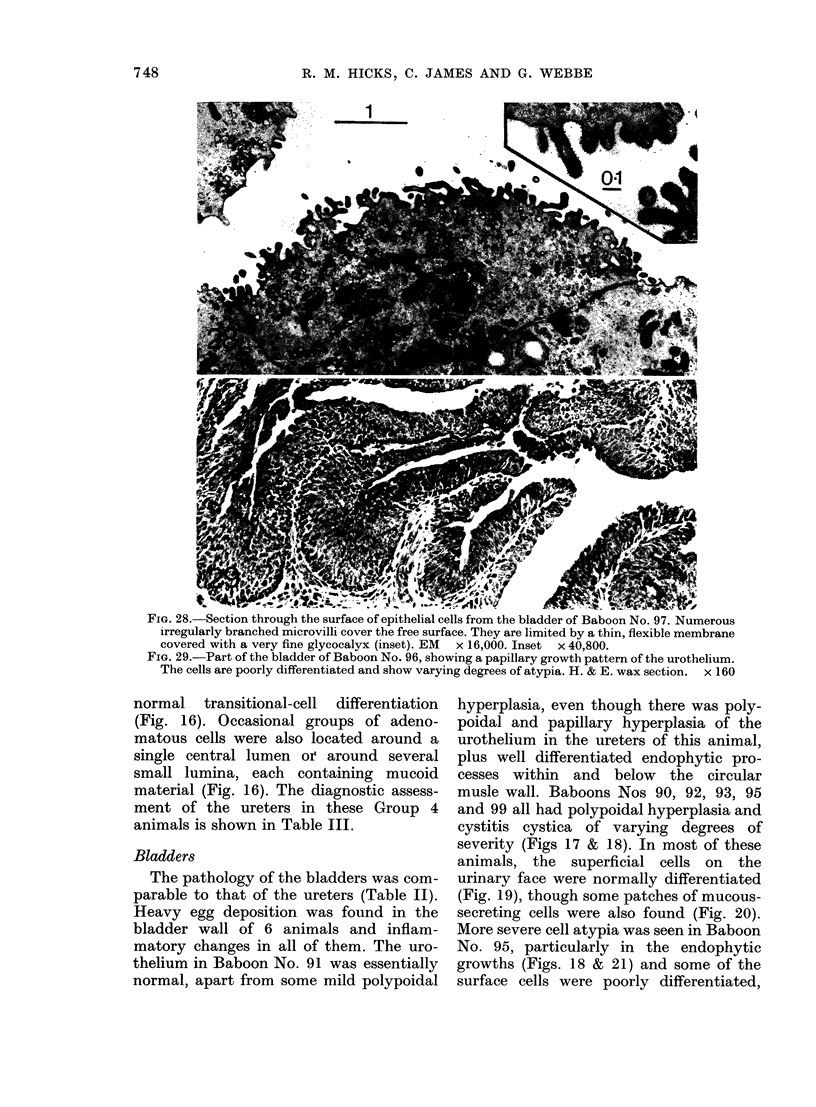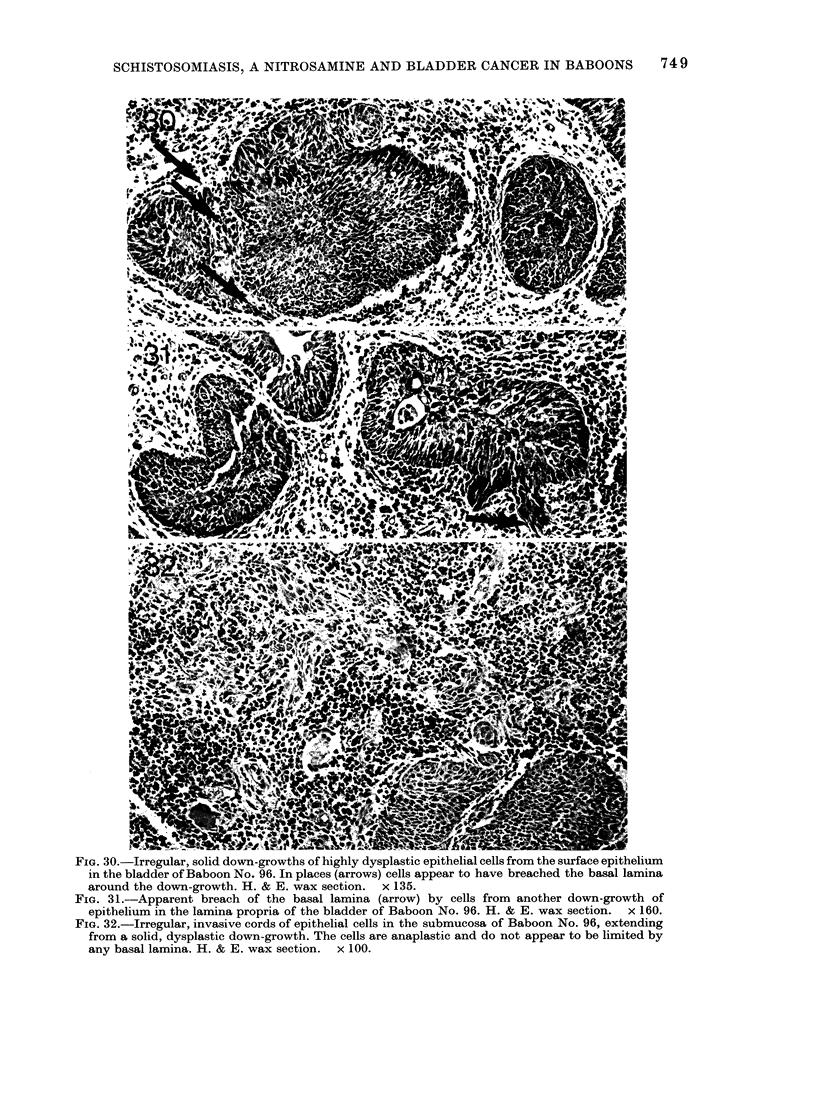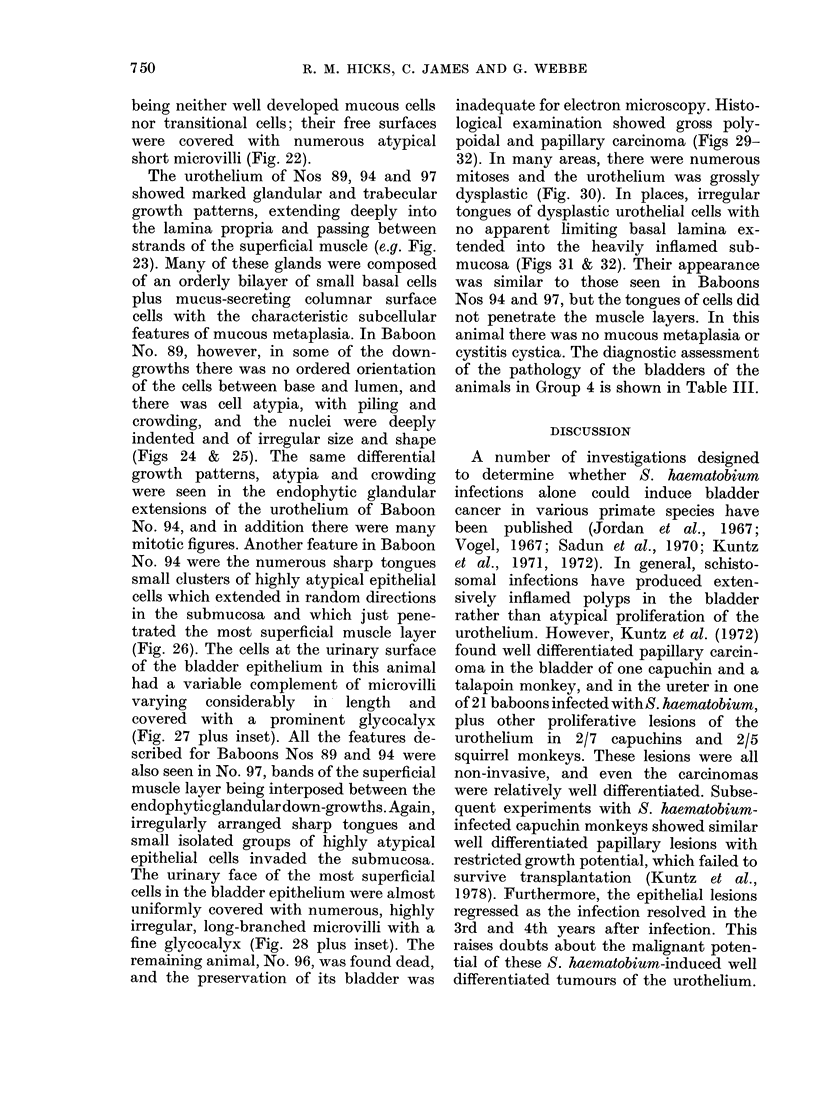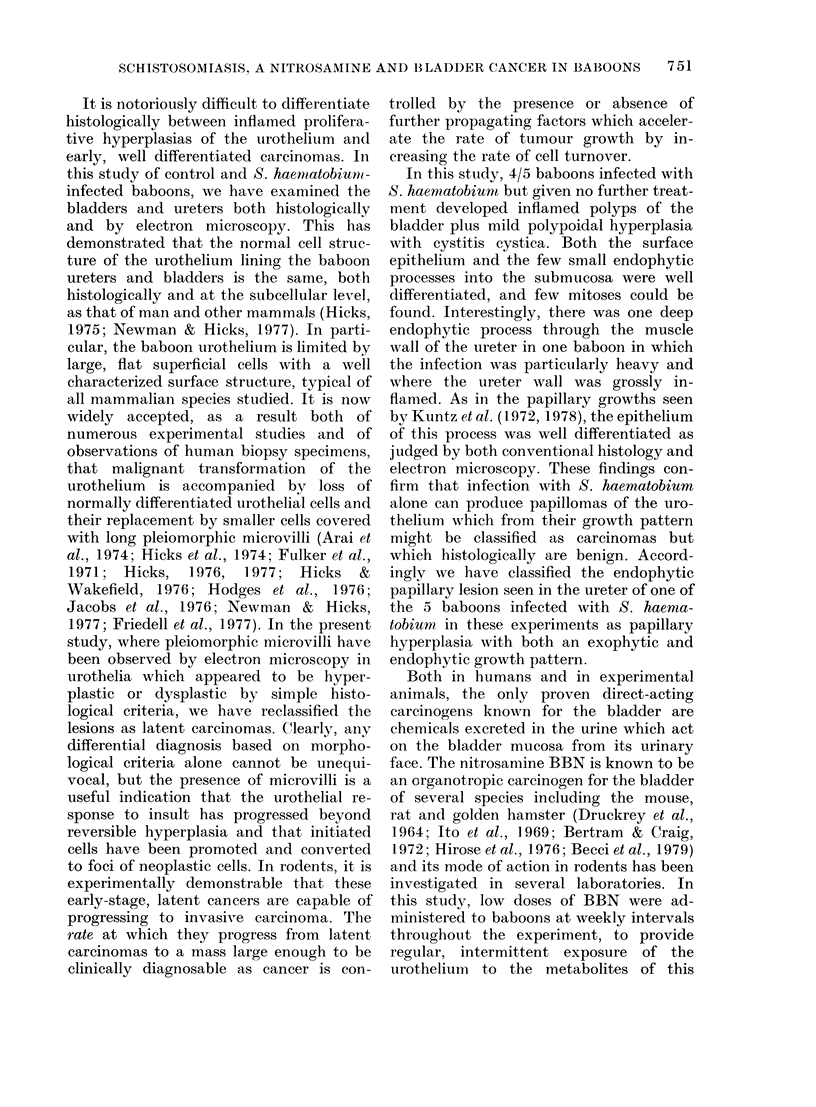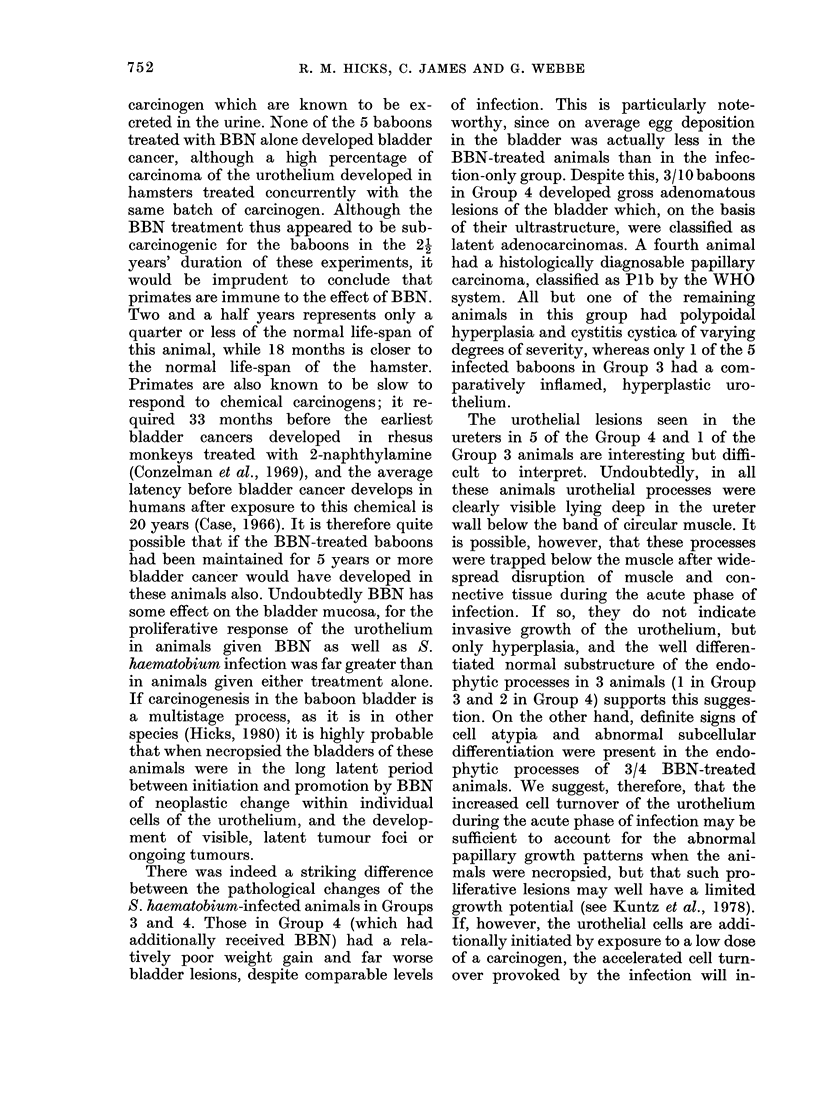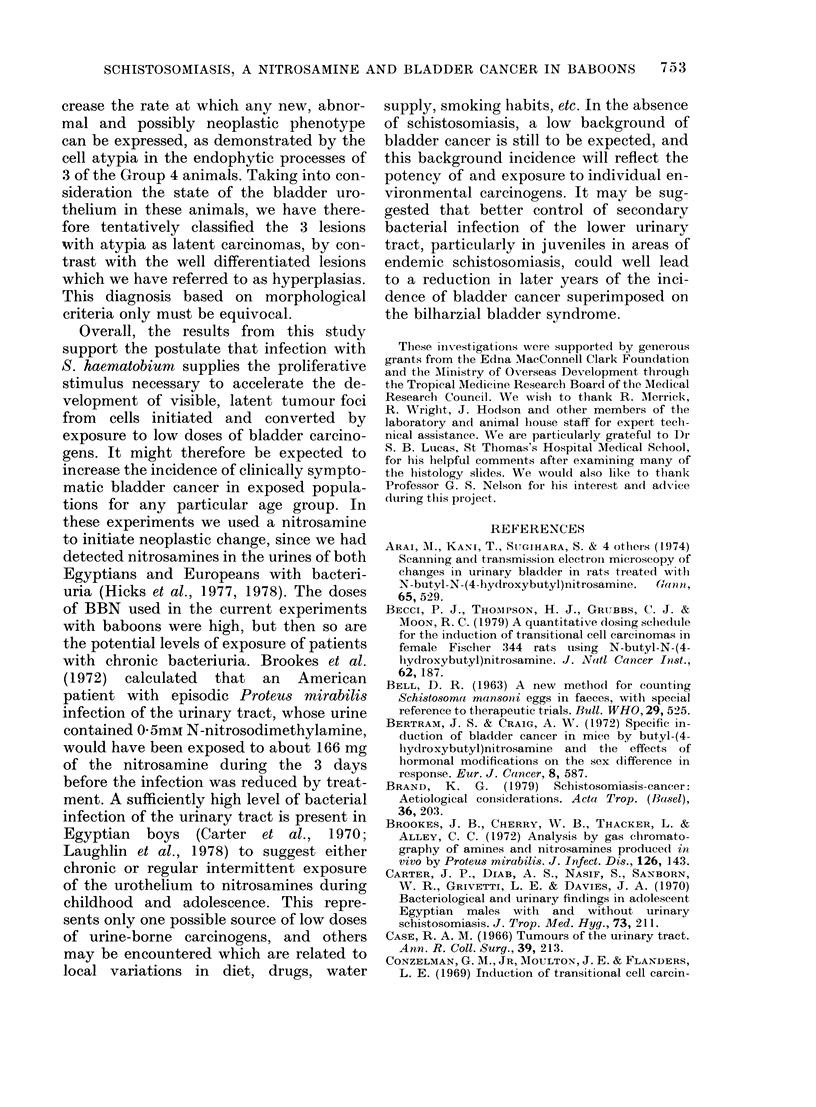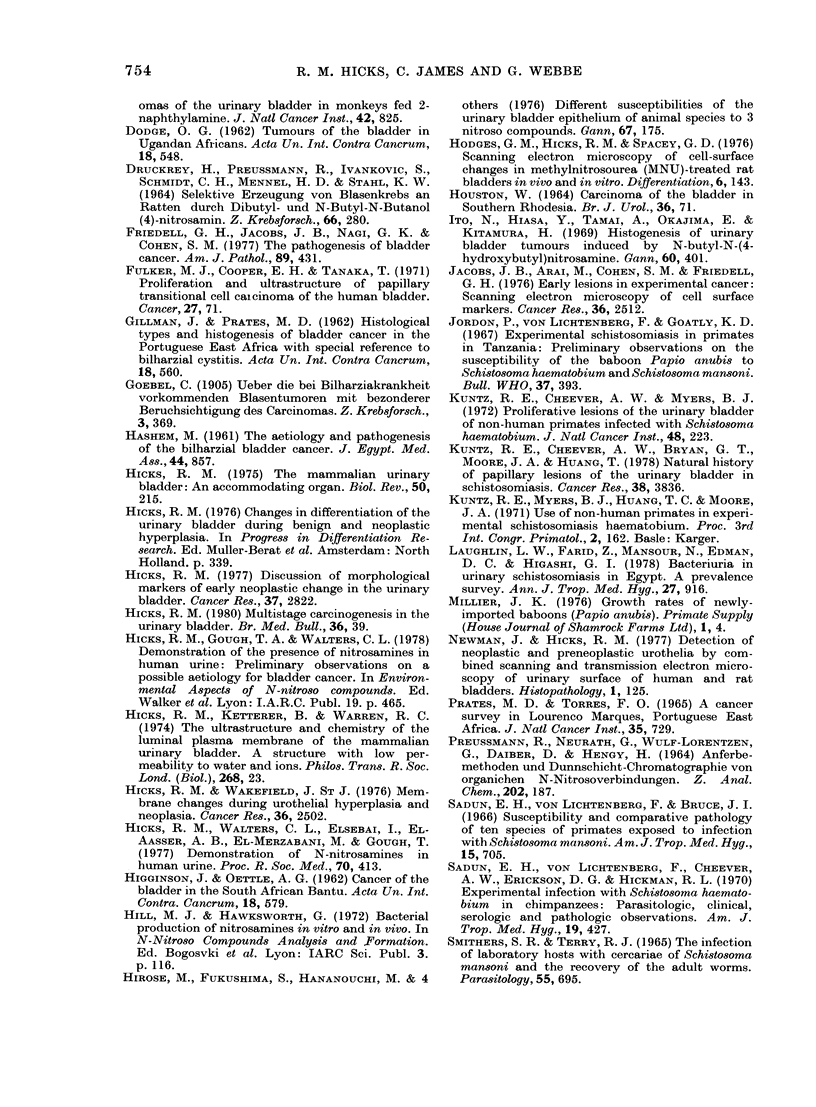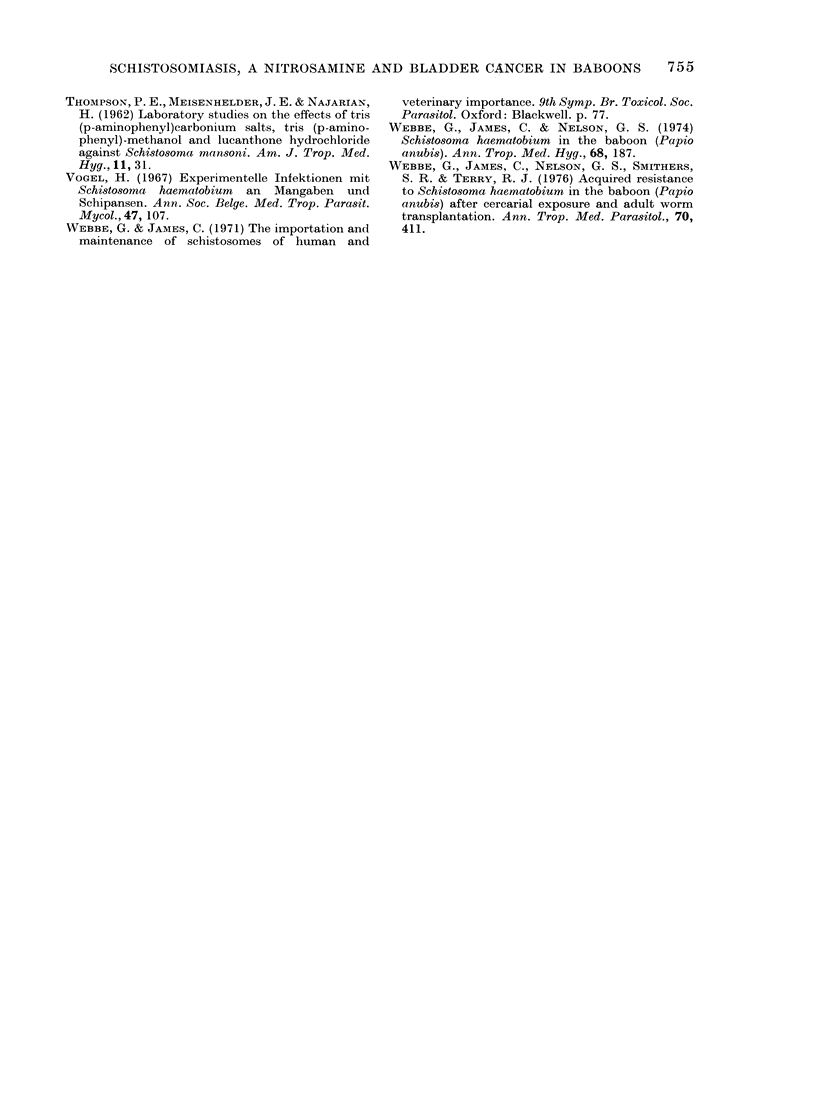# Effect of Schistosoma haematobium and N-butyl-N-(4-hydroxybutyl)nitrosamine on the development of urothelial neoplasia in the baboon

**DOI:** 10.1038/bjc.1980.308

**Published:** 1980-11

**Authors:** R. M. Hicks, C. James, G. Webbe

## Abstract

**Images:**


*Br. J. Cancer* (1980) **42**, 730

### EFFECT OF *SCHISTOSOMA HAEMATOBIUM* AND N-BUTYL-N-(4-HYDROXYBUTYL)NITROSAMINE ON THE DEVELOPMENT OF UROTHELIAL NEOPLASIA IN THE BABOON

R. M. HICKS*, C. JAMES† AND G. WEBBE†

*From the *Department of Cell Pathology, Middlesex Hospital Medical School, London W1P 7LD and †Department of Medical Helminthology, London School of Hygiene and Tropical Medicine, London W.C.1*

Received 27 April 1980 Accepted 13 August 1980

**Summary.—Experiments were conducted to determine whether bladder cancer would develop in primates (*Papio* sp.) infected with *S. haematobium* and concurrently exposed to low initiating doses of the bladder carcinogen N-butyl-N-(4-hydroxybutyl)nitrosamine (BBN). To control for the systemic effects of schistosomiasis, 5 baboons were infected with *S. mansoni*, which does not lay its eggs in the bladder wall; to control for the effect of the carcinogen alone, 5 others were treated with BBN alone at the rate of 5 or 50 mg/kg per week for the duration of the experiment. Five animals were infected with S. haematobium and had no further treatment, and the main experimental group of 10 baboons was infected with *S. haematobium* and also treated weekly with 5 mg/kg BBN for up to 2½ years. Four of the 10 animals in the last group, but none in the three control groups developed neoplastic disease of the urothelium. Four animals with *S. haematobium* plus BBN treatment developed *in situ* carcinoma in the bladder (3 latent adenomatous lesions and 1 more advanced papillary tumour) and 2 of these animals plus 1 other had slightly dysplastic urothelial endophytic papillary growths of the ureter which penetrated the muscle layer. By contrast, none of the control animals developed urothelial carcinomas, though 4/5 of those with *S. haematobium* infection alone had inflamed bladders with polypoid lesions, and one individual had endophytic papillary hyperplasia of the ureter. The animals were killed after 2½ years while still relatively immature or adolescent, and it is possible that had they been allowed to survive longer some of the BBN-only group would have developed bladder cancer, and more of the latent lesions seen in the BBN+schistosomiasis group would have progressed to invasive carcinoma. It is postulated that, in this model for human bilharzial bladder cancer, schistosomiasis supplies the proliferative stimulus necessary to accelerate cancer growth from latent tumour foci produced by exposure to low doses of the bladder carcinogen. In areas of endemic schistosomiasis, carcinogenesis might be initiated, for example, by low doses of nitrosamines produced in the urinary tract during bouts of bacteriuria.**

THE CLINICAL and pathological consequences of *S. haematobium* infection vary, and may be related directly to the prevalence and intensity of the infection. Thus in Egypt and Mozambique, where both prevalence and intensity are high, the close association of schistosomiasis and bladder cancer has been emphasized by many workers (Goebel, 1905; Hashem, 1961; Gillman & Prates, 1962; Prates & Torres, 1965; Brand, 1979) whereas in other areas with a lower endemicity the association is less evident (*e.g.* Higginson & Oettle, 1962; Dodge, 1962; Houston, 1964). Another possibility is that the connection between schistosomiasis and bladder cancer is more indirect.

There is now good experimental evidence from animal models that the biogenesis of bladder cancer is a multistage phenomenon, involving initiation by a carcinogen followed by promotion and propagation of tumour growth by other factors which are not necessarily carcinogens *per se* (Hicks, 1980). All proven bladder carcinogens identified so far are chemicals which act on the urothelium from its urinary face, and they include a number of N-nitroso compounds which may be formed in the urine during bouts of bacterial infection of the urinary tract (Hill & Hawksworth, 1972; Brookes *et al.*, 1972). Preliminary investigations demonstrated that nitrosamines are indeed formed in the urine in some individuals in Egypt who have both *S. haematobium* and bacterial infection of the urinary tract (Hicks *et al.*, 1977, 1978).

In Egypt, the incidence of bacteriuria is generally low in adults, but a significantly high level of infection (10–37%) was found in village boys and was associated more with poor standards of hygiene than with *S. haematobium* infection (Carter *et al.*, 1970). More recently, a 5·1% incidence of bacteriuria was found in 390 Egyptian boys aged 5–16 years in regions of endemic *S. haematobium* infection, and this was 10 times that in areas non-endemic for schistosomiasis (Laughlin *et al.*, 1978). It is thus theoretically possible that the urothelia of a significant percentage of juveniles are intermittently exposed to nitrosamines during bouts of bacterial cystitis. Bladder cancer has a long, clinically "silent" asymptomatic latent period, and in Egypt the peak prevalence (*i.e.* diagnosis) is in middle age: $50 \pm 5$ years. It is possible that urine-borne nitrosamines produced in childhood or adolescence during episodes of bacteriuria may initiate neoplastic change in the urothelium, and that in areas where schistosomiasis is endemic the development of bladder cancer is subsequently accelerated by chronic irritation of the bladder wall by erupting or calcified ova.

One attraction of this theory is that it accounts satisfactorily for the observed association of high bladder-cancer incidence with *S. haematobium* but not with *S. mansoni* infections. *S. mansoni*, unlike *S. haematobium*, does not deposit its eggs in the bladder wall, and thus does not provide a direct irritant stimulus to accelerate the development of any previously induced neoplastic lesion. On the other hand, the adults of both species live within the host's vascular system and, if carcinogens were excreted or secreted by the worms *per se*, the bladder should be equally at risk from blood-borne and/or urine-borne carcinogens in both *S. haematobium* and *S. mansoni* infections. A further argument in favour of the above theory, involving initiation of tumour growth by a carcinogen from an alternative source, is that though both *S. haematobium* and *S. mansoni* deposit their eggs in the submucosa of the bowel, there is no elevation of cancer of the bowel associated with these infections. Only bladder cancer is elevated, and that only in the *S. haematobium*-infected population, where the ova are in the bladder wall in a position to promote tumour growth if the urothelium has been initiated by some environmental, bladder-specific carcinogen.

The work reported here was designed to investigate whether in baboons the process of carcinogenesis initiated in the urinary bladder by low, threshold doses of a urine-borne carcinogen is accelerated by the proliferative changes in the urothelium which develop in response to *S. haematobium* infection. It was not designed as a classic initiation/promotion trial in which the initiating agent is given first and then later is followed by a promoting regime. Instead, in order to simulate the human condition, the *S. haematobium* infection was established first and small pulses of nitrosamines were administered over a long period of time. There was thus concurrent exposure of the urothelium to schistosomiasis and to the carcinogenic urinary metabolites. In this system, the carcinogen could theoretically initiate neoplastic change at the cellular level and also convert initiated cells to small foci of latent tumour cells. It was postulated that the schistosomiasis might supply the proliferative stimulus necessary to accelerate the development of ongoing tumours from such latent foci.

### MATERIALS AND METHODS

*Carcinogen*

N - butyl - N(4 - hydroxybutyl)nitrosamine (BBN) was synthesized for this experiment by Cambrian Chemicals Ltd., Beddington Farm Road, Croydon, by the method of Druckrey *et al.* (1964). The redistilled BBN was shown to be chromatographically pure by the method of Preussmann *et al.* (1964). It was stored undiluted in the dark at −20°C, and used as required for injection as a 25 or 2·5% solution in saline.

*Animals*

Twenty-five young male baboons (*Papio* sp.) weighing 3–8 kg on arrival were divided into 5 experimental treatment groups as follows:

| Group | Animal identity | Treatment |
|---|---|---|
| 1 | 104, 107, 111, 112, 113 | 1,000 cercariae of *S. mansoni* |
| 2 | 84, 85, 86 | 50 mg/kg BBN weekly |
| | 87, 88 | 5 mg/kg BBN weekly |
| 3 | 98, 100, 101, 102, 103 | 1,000 cercariae of *S. haematobium* |
| 4 | 89–93, 94–97, 99 | 5 mg/kg BBN + *S. haematobium* |

*Group* 1.—The 5 animals in this group were infected with *S. mansoni* using the abdominal-pouch method illustrated by Webbe & James (1971). The animals were necropsied 7–8 months after infection.

*Group* 2.—Three baboons in this group were given i.m. injections of 50 mg/kg BBN and the other 2 were given one tenth of this dose, namely 5 mg/kg BBN per week.

*Group* 3.—The 5 baboons in this group were infected with the Ghanaian strain of *S. haematobium.*

*Group* 4.—There were 10 animals in this group, infected as for Group 3 with *S. haematobium* and also given weekly injections of the lower dose of 5 mg/kg BBN. The injections were started one week after infection with *S. haematobium* and continued until necropsy.

The baboons were kept for 2½ years before necropsy, with the exception of 2 animals, Nos 94 and 95, which were necropsied prematurely at 12 and 14 months respectively, and No. 96 which died during the acute stage, 3 months after infection.

After infection, the cercariae develop into sexually mature adults in 8–10 weeks, and eggs are excreted in the faeces and urine from *S. haematobium* animals from then on (Webbe *et al.*, 1974). Both the urinary egg count and the faecal egg count reflect the progress and intensity of the infection, and give some indication of the probable onset of inflammatory hyperplasia of the urothelium in response to the infection. The progress of the infection in Group 3 animals was monitored throughout the experiment by weekly urinary and faecal egg counts, using the Bell filtration technique (Bell, 1963). The faeces and urines of Group 4 animals were also sporadically tested for the presence of eggs, but regular handling of the excreta was avoided because of possible contact with excreted carcinogen. Egg-excretion graphs were drawn, using the 3-point moving average method (Thompson *et al.*, 1962). All baboons were weighed monthly, and at the same time blood samples were taken and packed cell volumes (PCV) determined. Three times a year the animals were examined radiologically with a Watson portable MX2 X-ray machine (Webbe *et al.*, 1974). At necropsy, the bladder was removed and instantly fixed for histology and electron microscopy. The baboons were then perfused using the method of Smithers & Terry (1965). Tissue egg counts and routine histopathology were performed as described by Webbe *et al.* (1976).

*Histology and electron microscopy of the bladder and ureters*

The bladder was inflated with phosphate-buffered formalin, then immediately opened and small areas, including the mucosal surface but discarding most of the muscle, were taken for electron microscopy. These were fixed in 1% osmium tetroxide buffered to pH 7·4 with 0·1M cacodylate for 1½ h at 4°C before being dehydrated through graded alcohols and embedded in Spurr resin. Sections were cut at $\sim 2$ μm and stained with toluidine blue for high-resolution light microscopy. Thin sections were cut on an LKB or a Porter-Blum microtome, contrast-stained with lead and uranyl salts and examined by transmission electron microscopy in a JEOL 100B or Phillips 200 electron microscope.

After removing areas for electron microscopy samples of the whole thickness of the bladder wall were taken for histology. These were fixed for a further 1–3 days in phosphate-buffered formalin before wax embedding, sectioning and staining with haematoxylin and eosin.

Ureters were immerse-fixed in formalin or osmium tetroxide and processed for histology or electron microscopy as described above.

### RESULTS

### *Parasitology*

The faecal egg output showed an early rise, then a fall after about one year (Fig. 1) which is characteristic for *S. haematobium* infections in the baboon (Webbe *et al.*, 1974). A summary of the infection data is given in Table I. As previously reported (Webbe *et al.*, 1974) the infection rate, expressed as percentage of worms recovered in relation to the number of penetrating cercariae, was very variable. Both the experimental Group 4 and the infection-control Group 3 included baboons with infection rates as low as 1–2%. One animal (No. 94) had an exceptionally high infection rate of 48% and had to be necropsied prematurely, and No. 96 died 3 months after infection, during the acute stage.

### *Body weight and packed cell volumes* (*PCV*)

The relative weight changes of baboons during the period of the experiment are shown in Figs 2–5. The normal average monthly gain for a 4kg baboon is 0·7 kg (Millier, 1976). This was not affected by carcinogen treatment alone or, with the exception of Baboon No. 101, by infection alone (Fig. 2). By contrast, the weight gain of baboons in experimental Group 4 receiving both BBN and infection was severely affected. Nos 94–99 actually lost weight (~16·7% in the first 10 months after patency) (Fig. 3), and only Nos 89 and 93 showed a normal weight gain.

The normal PCV value of 40–45 remained constant in Group 2, despite BBN treatment, but showed a severe depression in all infected animals in Groups 3 and 4, coincident with the time of maximum egg output (Figs 2 & 3). As the infection became chronic, the PCV values rose with the exception of that of the heavily infected animal No. 94 which was necropsied early with a PCV of 11.

TABLE I.—*Baboons infected with* S. haematobium (1000 *cercariae/kg*). *A summary of parasitological data*

| Baboon | | | No. cercs. pene-trated | Duration of infect. (months) | Worm numbers | | | Infection rate (%) | Mean tissue eggs/g | Bladder tissue eggs/g |
|---|---|---|---|---|---|---|---|---|---|---|
| | Type | Weight at necropsy (kg) | | | ♂ | ♀ | Total | | | |
| BBN (5 mg/kg/wk) and *S. haematobium* | | | | | | | | | | |
| 89 | Hamadryas | 11·4 | 5300 | 30 | 143 | 72 | 215 | 4·1 | 100* | — |
| 90 | Olive | 8·3 | 7500 | 30 | 391 | 267 | 658 | 8·8 | 1700* | — |
| 91 | Olive | 7·2 | 6400 | 30 | 78 | 40 | 118 | 1·8 | 900* | — |
| 92 | Olive | 8·6 | 6500 | 30 | 389 | 208 | 597 | 9·2 | 250 | 0 |
| 93 | Olive | 9·9 | 5300 | 30 | 373 | 119 | 492 | 9·3 | 500 | 10 |
| 94 | Olive | 3·6 | 5000 | 11 | 1242 | 1157 | 2399 | 48·0 | 19000* | — |
| 95 | Olive | 4·1 | 5100 | 14 | 340 | 230 | 570 | 11·2 | 5000* | — |
| 95 | Yellow | 4·4 | 4750 | 3 | 433 | 236 | 669 | 14·1 | 2100 | 2600 |
| 97 | Olive | 4·1 | 5000 | 30 | 408 | 320 | 728 | 14·5 | 4800* | — |
| 99 | Olive | 3·4 | 5050 | 25 | 280 | 159 | 439 | 8·7 | 1900 | — |
| *S. haematobium* only | | | | | | | | | | |
| 98 | Olive | 11·0 | 6300 | 30 | 328 | 313 | 641 | 10·2 | 700 | 130 |
| 100 | Olive | 10·2 | 4800 | 31 | 258 | 81 | 339 | 7·1 | 1200 | 3700 |
| 101 | Hamadryas | 5·4 | 5400 | 30 | 32 | 19 | 51 | 0·9 | 40 | 60 |
| 102 | Hamadryas | 15·2 | 9100 | 29 | 154 | 154 | 308 | 3·4 | 200 | 40 |
| 103 | Hamadryas | 12·3 | 9200 | 30 | 95 | 23 | 118 | 1·3 | 0·8 | 0 |

* Excluding bladder.

*Radiology and gross pathology*

The gross pathological changes of animals in Control Group 1 were typical of *S. mansoni* infection (Sadun *et al.*, 1966). These were spotting and brown discoloration of the liver and spleen. Extensive haemorrhages were seen in the small and large intestines. The bladder and ureters of these animals were, however, essentially normal.

FIG. 1.—Bi-monthly egg-excretion patterns in the *S. haematobium*-infected baboons (Group 3).

FIG. 2.—Weight and PCV in the *S. haematobium*-infected baboons (Group 3).

FIG. 3.—Weight and PCV in 5 of the *S. haematobium*-infected baboons receiving 5 mg/kg BBN (Group 4).

The radiology and gross pathological lesions of the BBN-treated, Group 2 animals were normal apart from slightly dilated ureters in Baboons Nos 84 and 86. The infected animals in Group 3 all had gross pathological changes typical of *S. haematobium* infection, including greatly dilated ureters, lung adhesions, enlarged lymph nodes, epididymitis, and sandy patches on the bladder wall (Webbe *et al.*, 1974). Similar lesions were seen in Group 4 animals receiving both the BBN and *S. haematobium*, but in addition the ureters were severely damaged in Baboons Nos 91–94, being nodular and granulomatous as well as grossly dilated. In Nos 95–97 the bladder wall was markedly thickened, and No. 97 also had an enlarged right kidney which appeared as hydronephrosis on the radiograph.

### *Histopathology of bladders and ureters*

*Groups* 1 *and* 2.—No evidence of infection of the bladder or ureters was seen in the *S. mansoni*-infected control baboons of Group 1. The urothelium remained histologically normal (Figs 4, 5 & 6) and demonstrated all the characteristic subcellular features of normal transitional epithelium described for other mammalian species (Hicks, 1975). The urothelium in both bladders and ureters of baboons in Group 2 treated for $2\frac{1}{2}$ years with BBN also remained normal and neither the higher (50 mg/kg/week) nor lower (5 mg/kg/week) dose produced any signs of cell death, hyperplasia, dysplasia or loss of differentiation at the histological or subcellular level (Figs 7 & 8). Concurrent experiments with the same batch of BBN administered to hamsters in their drinking water at doses of 17·5 mg/kg week and 175 mg/kg/week had, by 18 months, produced bladder cancer in about one third and three quarters of the treated animals, respectively.

*Group* 3.—The bladders of baboons in Group 3 (*S. haematobium* infection only)

FIG. 4.—Partof the bladder wall of a *S. mansoni*-infected baboon (No. 112) to illustrate normal baboon bladder. No eggs are deposited in the bladder wall, and the urothelium is of normal thickness and is normally differentiated. H. & E. wax section. × 64.

FIG. 5.—A thin section through the luminal membrane of a superficial cell from the urothelium of a control *S. mansoni*-infected baboon. The asymmetrically thickened membrane (m) which limits the cell surface and invaginates as vesicles (v) into the apical cytoplasm when the bladder contracts, is a marker for normal differentiation in mammalian urothelia. EM × 160,000.

FIG. 6.—Cells at the surface of the normal urothelium of a control *S. mansoni*-infected baboon. The large, superficial cells have a scalloped profile, and contain vesicles in their apical cytoplasm formed of the thick specialized membrane shown at higher magnification in Fig. 5. The subcellular appearance of these cells is characteristic of normal differentiation in this tissue. EM × 4400.

FIG. 7.—A section through the ureter of Baboon No. 85 treated with weekly injections of 50 mg/kg BBN. The urothelium is of normal thickness and normally differentiated. H. & E. wax section. × 64.

FIG. 8.—Normally differentiated urothelium lining the bladder of Baboon No. 86 treated with 50 mg/kg BBN. The large surface cells indicate normal maturation of the urothelium. Toluidine-blue-stained, epon-embedded section. × 163.

FIG. 9.—Section through part of the bladder wall of Baboon No. 101, showing mild polypoid hyperplasia with cystitis cystica. The urothelium is slightly thickened but appears to be well-differentiated. Inflammatory cells are present in the lamina propria. H. & E. wax section. × 88.

FIG. 10.—A grossly inflamed polyp in the ureter of the *S. haematobium*-infected Baboon No. 98. *S. haematobium* ova are deposited in the submucosa and the urothelium is invaginated as a number of processes, each containing a lumen in contact with the surface out of the plane of this section. H. & E. × 88.

FIG. 11.—Another section through a ureter of Baboon No. 98. This field also shows deep invaginations of the surface urothelium, each with its lumen, and one of these processes is seen to lie below the circular muscle (arrow). The urothelium lining both the invaginated processes and the luminal face of the ureter appears to be well-differentiated. Toluidine-blue-stained, epon-embedded section. × 136.

FIG. 12.—This section shows polypoidal hyperplasia with cystitis cystica in the bladder of Baboon No. 98 infected with *S. haematobium*. The urothelium is well-differentiated and not much thicker than normal. Part of a lymphoid follicle is shown in this field. Toluidine-blue-stained, epon-embedded section. × 88.

FIG. 13.—Thin section through part of the urothelium lining the bladder of Baboon No. 98. The cells are normally differentiated (*cf*. Fig. 6) and are limited on their luminal face by the characteristic, angular luminal membrane. EM × 4000.

FIG. 14.—Cross-section of one ureter in the *S. haematobium*-infected, BBN-treated Baboon No. 95, showing hyperplasia of the urothelium. Two well differentiated urothelial processes (arrows) are located within or below the circular muscle. H. & E. wax section. ×64.

FIG. 15.—A more dysplastic urothelial process below the circular muscle in the ureter of Baboon No. 95. The cells in the process are irregular in shape and size and are smaller than those of the surface epithelium. Their growth pattern is irregular and there is no sign of normal maturation or differentiation. Toluidine-blue-stained, epon-embedded section. ×144.

FIG. 16.—This section shows part of a displastic urothelial process below the muscle in the ureter of a *S. haematobium*-infected, BBN-treated baboon. This process had several small, irregular lumina, each of which was surrounded by cells which varied considerably in their staining properties. In this field the cells have an irergular growth pattern, variably sized and shaped nuclei, and have none of the markers of normal urothelial cell differentiation. The apical cytoplasm of the cells adjacent to the lumen contain mucous-secreting granules and the lumen contains mucoid material. EM × 5400.

were mostly heavily loaded with ova both deep in the wall and in the lamina propria. The infection was associated with the characteristic inflammatory response by the host, and numerous polymorphs, scattered lymphocytes and lymphoid follicles were seen in the tissues. In one individual, #103, the urothelium was normal despite considerable inflammation, but no ova were found in the submucosa. Three animals, Nos 100, 101 and 102, had mild cystitis cystica and polypoid hyperplasia (Fig. 9) with heavily inflamed lamina propria between the epithelial surfaces. In these animals the mucosa was slightly thickened but well differentiated, there were no down-growths through the muscle and the few small endophytic processes into the submucosa mainly had a visible lumen. The ureters were minimally infected and their urothelium was normal. The ureters of the 5th animal, No. 98, were severely infected, grossly inflamed with heavy egg deposition and marked polypoidal hyperplasia of the urothelium showing mild dysplasia (Fig. 10). One small endophytic process of urothelium with a lumen had penetrated the muscle in the wall of one ureter, but the cells in this area were well differentiated (Fig. 11). The bladder of this animal was also severely infected and inflamed, and again there was polypoidal hyperplasia and cystitis cystica (Fig. 12). However, the bladder urothelium was well differentiated, there was no dysplasia and electron-microscopical examination showed the surface cells to be normal, slightly immature superficial cells (Fig. 13). The histopathology of Group 3 bladders and ureters is summarized in Table II.

The urothelia lining the ureters and bladders of animals in Group 4, which were infected with *S. haematobium* and given weekly injections of the lower-dose BBN (5 mg/kg), showed more florid proliferative changes and more dysplastic histology than animals in Group 3, which were infected with *S. haematobium* but did not receive the carcinogen. Table II summarizes the histopathological findings in the ureters and bladders of these animals in relation to egg deposition and inflammation of the walls of the ureters and bladder.

FIG. 17.—Polypoidal hyperplasia and cystitis cystica of the urothelium in the *S. haematobium*-infected, BBN-treated Baboon No. 92. The urothelial processes do not penetrate the superficial muscle and the urothelium is relatively well-differentiated. H. & E. wax-embedded section. × 64.

FIG. 18.—Polypoidal hyperplasia and cystitis cystica of the urothelium in Baboon No. 95, infected with *S. haematobium* and treated with BBN. In some areas (arrows) the epithelium is showing an abnormal differential growth pattern and dysplasia. Toluidine-blue-stained, epon-embedded section. × 160.

TABLE II.—*Growth pattern of urothelium*

| Baboon No. | | State of submucosa: Density of *S. haematobium* ova | State of submucosa: Inflammatory response | Growth pattern of urothelium: Normal Flat | Hyperplastic: Flat | Hyperplastic: Polypoidal | Hyperplastic: Cystitis cystica | Hyperplastic: Calcified nodules | Hyperplastic: Glandular trabeculae | Hyperplastic: Papillary | Hyperplastic: Through superficial muscle | Histology of urothelium: Normal | Histology of urothelium: Dysplasia | Histology of urothelium: Mucous metaplasia | Histology of urothelium: Mitoses | Histology of urothelium: Loss of basal lamina |
|---|---|---|---|---|---|---|---|---|---|---|---|---|---|---|---|---|
| Group 1 and Group 2 animals | | | | | | | | | | | | | | | | |
| 104, 107, 111–113, 84–88 | Bladder | | | + | | | | | | | | + | | | | |
| | Ureter | | | + | | | | | | | | + | | | | |
| Group 3 animals | | | | | | | | | | | | | | | | |
| 98 | Bladder | +++++ | +++ | | | + | + | | | | | + | | | | |
| | Ureter | +++++ | ++++ | | | ++ | + | | | | + | + | + | + | | |
| 100 | Bladder | ++++ | +++ | | | + | + | | | | | + | | | | |
| | Ureter | + | | + | | | | | | | | + | | | | |
| 101 | Bladder | ++++ | +++ | | | + | + | | | | | + | | | | |
| | Ureter | | | + | | | | | | | | + | | | | |
| 102 | Bladder | +++ | +++ | | | + | + | | | | | + | + | | | |
| | Ureter | + | | + | | | | | | | | + | | | | |
| 103 | Bladder | | +++ | + | | + | + | | | | | + | | | | |
| | Ureter | + | + | + | | | | | | | | + | | | | |
| Group 4 animals | | | | | | | | | | | | | | | | |
| 89 | Bladder | +++ | +++ | | | ++ | ++ | + | +++ | + | + | | ++ | + | + | |
| | Ureter | ++ | ++ | | | + | + | | | + | ++ | + | + | + | | |
| 90 | Bladder | + | ++ | | | | + | | | + | | + | | + | | |
| | Ureter | + | + | | + | | | | | | | + | | | | |
| 91 | Bladder | + | + | + | | + | | | | | | + | | | | |
| | Ureter | ++ | +++ | | | + | | | | + | + | + | | | | |
| 92 | Bladder | + | + | | | + | + | + | | | | + | | + | | |
| | Ureter | ++ | +++ | | | + | + | + | | + | | + | | + | | |
| 93 | Bladder | +++ | ++ | | | + | + | + | | | | | | + | | |
| | Ureter | + | ++ | | | + | | | | + | ++ | + | | | | |
| 94 | Bladder | +++ | +++ | | | ++ | ++ | | +++ | | + | | ++ | ++ | ++ | |
| | Ureter | +++ | +++ | | | ++ | ++ | | | + | | | | ++ | | |
| 95 | Bladder | +++ | +++ | | | +++ | ++ | | ++ | + | | | ++ | ++ | | |
| | Ureter | ++++ | +++ | | | + | + | | | + | +++ | + | + | ++ | | |
| 96 | Bladder | +++++ | +++ | | | +++ | | | | +++ | | | +++ | | ++ | + |
| 97 | Bladder | +++ | ++++ | | | + | ++ | ++ | +++ | | + | | +++ | ++ | + | |
| | Ureter | ++++ | ++ | | | | | | | | ++ | | ++ | ++ | | |
| 99 | Bladder | ++ | ++ | | | + | + | + | | + | | + | | + | | |
| | Ureter | | ++ | + | | | | | | | | + | | | | |

*Ureters*

The ureters of Baboon No. 96, which was found dead, were not sampled. Baboon No. 99 was the only animal in which the ureteral urothelium was mainly of normal thickness and well differentiated. No schistosomal ova were found in the ureters of this animal, even though moderate egg deposition accompanied by reactive hyperplasia of the urothelium was found in its bladder. In the ureters of one other individual (No. 90) there was a thickened hyperplastic urothelium but a normal growth pattern with normal cell differentiation; very few ova were found in the submucosa, though the ureters were slightly inflamed. The remaining 7 animals all had moderate to severe inflammation of the ureters, variable numbers of eggs being deposited deep in the ureter wall and sometimes superficially in the submucosa. In 5 animals (Nos 89, 91, 93, 95 and 97) there were endophytic processes of urothelium clearly located within or beneath the circular muscle in the wall of the ureter. In two of these (Nos 91 and 93) cell differentiation appeared normal, and comparable to that in the *S. haematobium* control animal shown in Fig. 11. One of the others No. 95) had numerous down-growths, many of which were well-differentiated (Fig. 14), but others were slightly dysplastic (Fig. 15). There were dysplastic down-growths in Nos 89 and 97 showing differential growth patterns, irregularity in size and shape of nuclei, and failure of

TABLE III.—*Diagnoses of urothelial histopathology in bladders and ureters of individual baboons*

| Baboon No. | | Diagnosis based on histology | Diagnosis based on subcellular structure plus histology |
|---|---|---|---|
| Groups 1 and 2 | | | |
| 104, 107, 111–113, 84–88 | Bladders and ureters | Normal | Normal |
| Group 3 | | | |
| 98 | Bladder | Marked polypoidal hyp. + cystitis cystica | Marked polypoidal hyp. + cystitis cystica |
| | Ureter | Marked polypoidal hyp. | Polypoidal + endophytic papillary hyp. |
| 100 | Bladder | Mild polypoidal hyp. + cystitis cystica | Mild polypoidal hyp. + cystitis cystica |
| | Ureter | Normal | Normal |
| 101 | Bladder | Mild polypoidal hyp. + cystitis cystica | Mild polypoidal hyp. + cystitis cystica |
| | Ureter | Normal | Normal |
| 102 | Bladder | Mild polypoidal hyp. + cystitis cystica | Mild polypoidal hyp. + cystitis cystica |
| | Ureter | Normal | Normal |
| 103 | Bladder | Normal | Normal |
| | Ureter | Normal | Normal |
| Group 4 | | | |
| 89 | Bladder | Adenomatous hyp. | *Latent adenocarcinoma P1b* |
| | Ureter | Polypoid hyp. | *Latent papillary carcinoma P1b* |
| 90 | Bladder | Hyp. | Hyp. + mucous metaplasia |
| | Ureter | Mild hyp. | Mild hyp. |
| 91 | Bladder | Mild polypoid hyp. | Mild polypoid hyp. |
| | Ureter | Polypoid hyp. | Exophytic papillary + endophytic hyp. |
| 92 | Bladder | Polypoid hyp. | Polypoid hyp. + cystitis cystica |
| | Ureter | Polypoid hyp. | Polypoid hyp. + cystitis cystica |
| 93 | Bladder | Polypoid hyp. + cystitis cystica | Polypoid hyp. + cystitis cystica |
| | Ureter | Polypoid hyp. | Papillary + endophytic hyp. |
| 94 | Bladder | Papillary + polypoid hyp. with mucous metaplasia | *Latent adenocarcinoma P1b* |
| | Ureter | Polypoid hyp. | Polypoid hyp. |
| 95 | Bladder | Gross polypoid hyp. + cystitis cystica | Atypical adenomatous hyp. with differential growth |
| | Ureter | Polypoid + papillary hyp. | *Latent papillary carcinoma P2* |
| 96 | Bladder | *Carcinoma* in situ *P1b* | (Not examined) |
| 97 | Bladder | Cystitis cystica | *Latent adenocarcinoma P1b* |
| | Ureter | Papillary hyp. | *Latent papillary carcinoma P2* |
| 99 | Bladder | Mild polypoidal hyp. + cystitis cystica | Mild polypoidal hyp. + cystitis cystica |
| | Ureter | Normal | Normal |

FIG. 19.—Normally differentiated urothelial cell in the bladder of Baboon No. 93. The subcellular structure of these cells is typical for normal urothelium. The cell to the right of the field is slightly immature but not abnormal. EM × 16,000.

FIG. 20.—Mucous-secreting cells at the free surface of the bladder epithelium in Baboon No. 93. The subcellular differentiation is typical of mucous metaplasia, and no normally differentiated urothelial cells are present in this field. EM × 12,600.

FIG. 21.—Thin section through an endophytic process of bladder urothelium found in Animal No. 95, infected with *S. haematobium* and treated with BBN. The cells are poorly differentiated and irregularly arranged; the nuclei are of varying shape and size. Normal urothelial cell features are absent. EM × 5400.

FIG. 22.—This field shows the surface of superficial cells in the bladder epithelium of Baboon No. 95. No normal urothelial cell differentiation; the cells are covered by numerous short, stubby, microvilli similar to but less regular than those seen on the surface of immature intermediate cells in a normal urothelium. EM × 12,600.

Fig. 23.—The glandular and trabecular epithelium lining the bladder of Baboon No. 89. The glands are mainly lined by a bilayer of densely staining basal cells and paler mucous-secreting cells; they extend deep into the submucosal connective tissue, parting and passing between strands of the superficial muscle layer (arrows). H. & E. wax section. × 54.

Fig. 24.—A glandular down-growth of epithelium in the bladder of Baboon No. 89. The epithelial cells are atypical, crowded, and piled into many layers between basal lamina and lumen. H. & E. wax section. × 135.

Fig. 25.—Thin section through the base of a glandular down-growth of epithelium in the bladder of Baboon No. 89. The cells vary in shape and size, and the normal orientation between basal lamina and surface is disturbed. The nuclei are abnormally indented and variable in appearance. EM × 6000.

FIG. 26.—Section through part of the bladder epithelium of Baboon No. 94. The luminal surface of the bladder is at the top left of the field, and irregular sharp tongues of epithelium (arrows) extend back from the surface into the submucosa. These randomly arranged processes are composed of very dysplastic, irregularly staining cells. Numerous live and calcified *S. haematobium* ova are present in the heavily inflamed submucosa. Toluidine-blue-stained epon section. × 144.

FIG. 27.—Section through a superficial cell at the surface of the epithelium lining the bladder of Baboon No. 94. The surface of the cells is covered with microvilli limited by a flexible membrane which has a prominent glycocalyx (inset). EM × 19,200. Inset × 54,000.

FIG. 28.—Section through the surface of epithelial cells from the bladder of Baboon No. 97. Numerous irregularly branched microvilli cover the free surface. They are limited by a thin, flexible membrane covered with a very fine glycocalyx (inset). EM × 16,000. Inset × 40,800.

FIG. 29.—Part of the bladder of Baboon No. 96, showing a papillary growth pattern of the urothelium. The cells are poorly differentiated and show varying degrees of atypia. H. & E. wax section. × 160

normal transitional-cell differentiation (Fig. 16). Occasional groups of adenomatous cells were also located around a single central lumen or around several small lumina, each containing mucoid material (Fig. 16). The diagnostic assessment of the ureters in these Group 4 animals is shown in Table III.

*Bladders*

The pathology of the bladders was comparable to that of the ureters (Table II). Heavy egg deposition was found in the bladder wall of 6 animals and inflammatory changes in all of them. The urothelium in Baboon No. 91 was essentially normal, apart from some mild polypoidal hyperplasia, even though there was polypoidal and papillary hyperplasia of the urothelium in the ureters of this animal, plus well differentiated endophytic processes within and below the circular musle wall. Baboons Nos 90, 92, 93, 95 and 99 all had polypoidal hyperplasia and cystitis cystica of varying degrees of severity (Figs 17 & 18). In most of these animals, the superficial cells on the urinary face were normally differentiated (Fig. 19), though some patches of mucous-secreting cells were also found (Fig. 20). More severe cell atypia was seen in Baboon No. 95, particularly in the endophytic growths (Figs. 18 & 21) and some of the surface cells were poorly differentiated,

FIG. 30.—Irregular, solid down-growths of highly dysplastic epithelial cells from the surface epithelium in the bladder of Baboon No. 96. In places (arrows) cells appear to have breached the basal lamina around the down-growth. H. & E. wax section. × 135.

FIG. 31.—Apparent breach of the basal lamina (arrow) by cells from another down-growth of epithelium in the lamina propria of the bladder of Baboon No. 96. H. & E. wax section. × 160.

FIG. 32.—Irregular, invasive cords of epithelial cells in the submucosa of Baboon No. 96, extending from a solid, dysplastic down-growth. The cells are anaplastic and do not appear to be limited by any basal lamina. H. & E. wax section. × 100.

being neither well developed mucous cells nor transitional cells; their free surfaces were covered with numerous atypical short microvilli (Fig. 22).

The urothelium of Nos 89, 94 and 97 showed marked glandular and trabecular growth patterns, extending deeply into the lamina propria and passing between strands of the superficial muscle (*e.g.* Fig. 23). Many of these glands were composed of an orderly bilayer of small basal cells plus mucus-secreting columnar surface cells with the characteristic subcellular features of mucous metaplasia. In Baboon No. 89, however, in some of the down-growths there was no ordered orientation of the cells between base and lumen, and there was cell atypia, with piling and crowding, and the nuclei were deeply indented and of irregular size and shape (Figs 24 & 25). The same differential growth patterns, atypia and crowding were seen in the endophytic glandular extensions of the urothelium of Baboon No. 94, and in addition there were many mitotic figures. Another feature in Baboon No. 94 were the numerous sharp tongues small clusters of highly atypical epithelial cells which extended in random directions in the submucosa and which just penetrated the most superficial muscle layer (Fig. 26). The cells at the urinary surface of the bladder epithelium in this animal had a variable complement of microvilli varying considerably in length and covered with a prominent glycocalyx (Fig. 27 plus inset). All the features described for Baboons Nos 89 and 94 were also seen in No. 97, bands of the superficial muscle layer being interposed between the endophytic glandular down-growths. Again, irregularly arranged sharp tongues and small isolated groups of highly atypical epithelial cells invaded the submucosa. The urinary face of the most superficial cells in the bladder epithelium were almost uniformly covered with numerous, highly irregular, long-branched microvilli with a fine glycocalyx (Fig. 28 plus inset). The remaining animal, No. 96, was found dead, and the preservation of its bladder was inadequate for electron microscopy. Histological examination showed gross polypoidal and papillary carcinoma (Figs 29–32). In many areas, there were numerous mitoses and the urothelium was grossly dysplastic (Fig. 30). In places, irregular tongues of dysplastic urothelial cells with no apparent limiting basal lamina extended into the heavily inflamed submucosa (Figs 31 & 32). Their appearance was similar to those seen in Baboons Nos 94 and 97, but the tongues of cells did not penetrate the muscle layers. In this animal there was no mucous metaplasia or cystitis cystica. The diagnostic assessment of the pathology of the bladders of the animals in Group 4 is shown in Table III.

### DISCUSSION

A number of investigations designed to determine whether *S. haematobium* infections alone could induce bladder cancer in various primate species have been published (Jordan *et al.*, 1967; Vogel, 1967; Sadun *et al.*, 1970; Kuntz *et al.*, 1971, 1972). In general, schistosomal infections have produced extensively inflamed polyps in the bladder rather than atypical proliferation of the urothelium. However, Kuntz *et al.* (1972) found well differentiated papillary carcinoma in the bladder of one capuchin and a talapoin monkey, and in the ureter in one of 21 baboons infected with *S. haematobium*, plus other proliferative lesions of the urothelium in 2/7 capuchins and 2/5 squirrel monkeys. These lesions were all non-invasive, and even the carcinomas were relatively well differentiated. Subsequent experiments with *S. haematobium*-infected capuchin monkeys showed similar well differentiated papillary lesions with restricted growth potential, which failed to survive transplantation (Kuntz *et al.*, 1978). Furthermore, the epithelial lesions regressed as the infection resolved in the 3rd and 4th years after infection. This raises doubts about the malignant potential of these *S. haematobium*-induced well differentiated tumours of the urothelium.

It is notoriously difficult to differentiate histologically between inflamed proliferative hyperplasias of the urothelium and early, well differentiated carcinomas. In this study of control and *S. haematobium*-infected baboons, we have examined the bladders and ureters both histologically and by electron microscopy. This has demonstrated that the normal cell structure of the urothelium lining the baboon ureters and bladders is the same, both histologically and at the subcellular level, as that of man and other mammals (Hicks, 1975; Newman & Hicks, 1977). In particular, the baboon urothelium is limited by large, flat superficial cells with a well characterized surface structure, typical of all mammalian species studied. It is now widely accepted, as a result both of numerous experimental studies and of observations of human biopsy specimens, that malignant transformation of the urothelium is accompanied by loss of normally differentiated urothelial cells and their replacement by smaller cells covered with long pleiomorphic microvilli (Arai *et al.*, 1974; Hicks *et al.*, 1974; Fulker *et al.*, 1971; Hicks, 1976, 1977; Hicks & Wakefield, 1976; Hodges *et al.*, 1976; Jacobs *et al.*, 1976; Newman & Hicks, 1977; Friedell *et al.*, 1977). In the present study, where pleiomorphic microvilli have been observed by electron microscopy in urothelia which appeared to be hyperplastic or dysplastic by simple histological criteria, we have reclassified the lesions as latent carcinomas. Clearly, any differential diagnosis based on morphological criteria alone cannot be unequivocal, but the presence of microvilli is a useful indication that the urothelial response to insult has progressed beyond reversible hyperplasia and that initiated cells have been promoted and converted to foci of neoplastic cells. In rodents, it is experimentally demonstrable that these early-stage, latent cancers are capable of progressing to invasive carcinoma. The *rate* at which they progress from latent carcinomas to a mass large enough to be clinically diagnosable as cancer is controlled by the presence or absence of further propagating factors which accelerate the rate of tumour growth by increasing the rate of cell turnover.

In this study, 4/5 baboons infected with *S. haematobium* but given no further treatment developed inflamed polyps of the bladder plus mild polypoidal hyperplasia with cystitis cystica. Both the surface epithelium and the few small endophytic processes into the submucosa were well differentiated, and few mitoses could be found. Interestingly, there was one deep endophytic process through the muscle wall of the ureter in one baboon in which the infection was particularly heavy and where the ureter wall was grossly inflamed. As in the papillary growths seen by Kuntz *et al.* (1972, 1978), the epithelium of this process was well differentiated as judged by both conventional histology and electron microscopy. These findings confirm that infection with *S. haematobium* alone can produce papillomas of the urothelium which from their growth pattern might be classified as carcinomas but which histologically are benign. Accordingly we have classified the endophytic papillary lesion seen in the ureter of one of the 5 baboons infected with *S. haematobium* in these experiments as papillary hyperplasia with both an exophytic and endophytic growth pattern.

Both in humans and in experimental animals, the only proven direct-acting carcinogens known for the bladder are chemicals excreted in the urine which act on the bladder mucosa from its urinary face. The nitrosamine BBN is known to be an organotropic carcinogen for the bladder of several species including the mouse, rat and golden hamster (Druckrey *et al.*, 1964; Ito *et al.*, 1969; Bertram & Craig, 1972; Hirose *et al.*, 1976; Becci *et al.*, 1979) and its mode of action in rodents has been investigated in several laboratories. In this study, low doses of BBN were administered to baboons at weekly intervals throughout the experiment, to provide regular, intermittent exposure of the urothelium to the metabolites of this carcinogen which are known to be excreted in the urine. None of the 5 baboons treated with BBN alone developed bladder cancer, although a high percentage of carcinoma of the urothelium developed in hamsters treated concurrently with the same batch of carcinogen. Although the BBN treatment thus appeared to be subcarcinogenic for the baboons in the $2\frac{1}{2}$ years' duration of these experiments, it would be imprudent to conclude that primates are immune to the effect of BBN. Two and a half years represents only a quarter or less of the normal life-span of this animal, while 18 months is closer to the normal life-span of the hamster. Primates are also known to be slow to respond to chemical carcinogens; it required 33 months before the earliest bladder cancers developed in rhesus monkeys treated with 2-naphthylamine (Conzelman *et al.*, 1969), and the average latency before bladder cancer develops in humans after exposure to this chemical is 20 years (Case, 1966). It is therefore quite possible that if the BBN-treated baboons had been maintained for 5 years or more bladder cancer would have developed in these animals also. Undoubtedly BBN has some effect on the bladder mucosa, for the proliferative response of the urothelium in animals given BBN as well as *S. haematobium* infection was far greater than in animals given either treatment alone. If carcinogenesis in the baboon bladder is a multistage process, as it is in other species (Hicks, 1980) it is highly probable that when necropsied the bladders of these animals were in the long latent period between initiation and promotion by BBN of neoplastic change within individual cells of the urothelium, and the development of visible, latent tumour foci or ongoing tumours.

There was indeed a striking difference between the pathological changes of the *S. haematobium*-infected animals in Groups 3 and 4. Those in Group 4 (which had additionally received BBN) had a relatively poor weight gain and far worse bladder lesions, despite comparable levels of infection. This is particularly noteworthy, since on average egg deposition in the bladder was actually less in the BBN-treated animals than in the infection-only group. Despite this, 3/10 baboons in Group 4 developed gross adenomatous lesions of the bladder which, on the basis of their ultrastructure, were classified as latent adenocarcinomas. A fourth animal had a histologically diagnosable papillary carcinoma, classified as P1b by the WHO system. All but one of the remaining animals in this group had polypoidal hyperplasia and cystitis cystica of varying degrees of severity, whereas only 1 of the 5 infected baboons in Group 3 had a comparatively inflamed, hyperplastic urothelium.

The urothelial lesions seen in the ureters in 5 of the Group 4 and 1 of the Group 3 animals are interesting but difficult to interpret. Undoubtedly, in all these animals urothelial processes were clearly visible lying deep in the ureter wall below the band of circular muscle. It is possible, however, that these processes were trapped below the muscle after widespread disruption of muscle and connective tissue during the acute phase of infection. If so, they do not indicate invasive growth of the urothelium, but only hyperplasia, and the well differentiated normal substructure of the endophytic processes in 3 animals (1 in Group 3 and 2 in Group 4) supports this suggestion. On the other hand, definite signs of cell atypia and abnormal subcellular differentiation were present in the endophytic processes of 3/4 BBN-treated animals. We suggest, therefore, that the increased cell turnover of the urothelium during the acute phase of infection may be sufficient to account for the abnormal papillary growth patterns when the animals were necropsied, but that such proliferative lesions may well have a limited growth potential (see Kuntz *et al.*, 1978). If, however, the urothelial cells are additionally initiated by exposure to a low dose of a carcinogen, the accelerated cell turnover provoked by the infection will increase the rate at which any new, abnormal and possibly neoplastic phenotype can be expressed, as demonstrated by the cell atypia in the endophytic processes of 3 of the Group 4 animals. Taking into consideration the state of the bladder urothelium in these animals, we have therefore tentatively classified the 3 lesions with atypia as latent carcinomas, by contrast with the well differentiated lesions which we have referred to as hyperplasias. This diagnosis based on morphological criteria only must be equivocal.

Overall, the results from this study support the postulate that infection with *S. haematobium* supplies the proliferative stimulus necessary to accelerate the development of visible, latent tumour foci from cells initiated and converted by exposure to low doses of bladder carcinogens. It might therefore be expected to increase the incidence of clinically symptomatic bladder cancer in exposed populations for any particular age group. In these experiments we used a nitrosamine to initiate neoplastic change, since we had detected nitrosamines in the urines of both Egyptians and Europeans with bacteriuria (Hicks *et al.*, 1977, 1978). The doses of BBN used in the current experiments with baboons were high, but then so are the potential levels of exposure of patients with chronic bacteriuria. Brookes *et al.* (1972) calculated that an American patient with episodic *Proteus mirabilis* infection of the urinary tract, whose urine contained 0·5mM N-nitrosodimethylamine, would have been exposed to about 166 mg of the nitrosamine during the 3 days before the infection was reduced by treatment. A sufficiently high level of bacterial infection of the urinary tract is present in Egyptian boys (Carter *et al.*, 1970; Laughlin *et al.*, 1978) to suggest either chronic or regular intermittent exposure of the urothelium to nitrosamines during childhood and adolescence. This represents only one possible source of low doses of urine-borne carcinogens, and others may be encountered which are related to local variations in diet, drugs, water supply, smoking habits, *etc.* In the absence of schistosomiasis, a low background of bladder cancer is still to be expected, and this background incidence will reflect the potency of and exposure to individual environmental carcinogens. It may be suggested that better control of secondary bacterial infection of the lower urinary tract, particularly in juveniles in areas of endemic schistosomiasis, could well lead to a reduction in later years of the incidence of bladder cancer superimposed on the bilharzial bladder syndrome.

These investigations were supported by generous grants from the Edna MacConnell Clark Foundation and the Ministry of Overseas Development through the Tropical Medicine Research Board of the Medical Research Council. We wish to thank R. Merrick, R. Wright, J. Hodson and other members of the laboratory and animal house staff for expert technical assistance. We are particularly grateful to Dr S. B. Lucas, St Thomas's Hospital Medical School, for his helpful comments after examining many of the histology slides. We would also like to thank Professor G. S. Nelson for his interest and advice during this project.

### REFERENCES

Arai, M., Kani, T., Sugihara, S. & 4 others (1974) Scanning and transmission electron microscopy of changes in urinary bladder in rats treated with N-butyl-N-(4-hydroxybutyl)nitrosamine. *Gann*, **65**, 529.

Becci, P. J., Thompson, H. J., Grubbs, C. J. & Moon, R. C. (1979) A quantitative dosing schedule for the induction of transitional cell carcinomas in female Fischer 344 rats using N-butyl-N-(4-hydroxybutyl)nitrosamine. *J. Natl Cancer Inst.*, **62**, 187.

Bell, D. R. (1963) A new method for counting *Schistosoma mansoni* eggs in faeces, with special reference to therapeutic trials. *Bull. WHO*, **29**, 525.

Bertram, J. S. & Craig, A. W. (1972) Specific induction of bladder cancer in mice by butyl-(4-hydroxybutyl)nitrosamine and the effects of hormonal modifications on the sex difference in response. *Eur. J. Cancer*, **8**, 587.

Brand, K. G. (1979) Schistosomiasis-cancer: Aetiological considerations. *Acta Trop.* (*Basel*), **36**, 203.

Brookes, J. B., Cherry, W. B., Thacker, L. & Alley, C. C. (1972) Analysis by gas chromatography of amines and nitrosamines produced *in vivo* by *Proteus mirabilis*. *J. Infect. Dis.*, **126**, 143.

Carter, J. P., Diab, A. S., Nasif, S., Sanborn, W. R., Grivetti, L. E. & Davies, J. A. (1970) Bacteriological and urinary findings in adolescent Egyptian males with and without urinary schistosomiasis. *J. Trop. Med. Hyg.*, **73**, 211.

Case, R. A. M. (1966) Tumours of the urinary tract. *Ann. R. Coll. Surg.*, **39**, 213.

Conzelman, G. M., Jr, Moulton, J. E. & Flanders, L. E. (1969) Induction of transitional cell carcinomas of the urinary bladder in monkeys fed 2-naphthylamine. *J. Natl Cancer Inst.*, **42**, 825.

DODGE, O. G. (1962) Tumours of the bladder in Ugandan Africans. *Acta Un. Int. Contra Cancrum*, **18**, 548.

DRUCKREY, H., PREUSSMANN, R., IVANKOVIC, S., SCHMIDT, C. H., MENNEL, H. D. & STAHL, K. W. (1964) Selektive Erzeugung von Blasenkrebs an Ratten durch Dibutyl- und N-Butyl-N-Butanol (4)-nitrosamin. *Z. Krebsforsch.*, **66**, 280.

FRIEDELL, G. H., JACOBS, J. B., NAGI, G. K. & COHEN, S. M. (1977) The pathogenesis of bladder cancer. *Am. J. Pathol.*, **89**, 431.

FULKER, M. J., COOPER, E. H. & TANAKA, T. (1971) Proliferation and ultrastructure of papillary transitional cell caıcinoma of the human bladder. *Cancer*, **27**, 71.

GILLMAN, J. & PRATES, M. D. (1962) Histological types and histogenesis of bladder cancer in the Portuguese East Africa with special reference to bilharzial cystitis. *Acta Un. Int. Contra Cancrum*, **18**, 560.

GOEBEL, C. (1905) Ueber die bei Bilharziakrankheit vorkommenden Blasentumoren mit bezonderer Beruchsichtigung des Carcinomas. *Z. Krebsforsch.*, **3**, 369.

HASHEM, M. (1961) The aetiology and pathogenesis of the bilharzial bladder cancer. *J. Egypt. Med. Ass.*, **44**, 857.

HICKS, R. M. (1975) The mammalian urinary bladder: An accommodating organ. *Biol. Rev.*, **50**, 215.

HICKS, R. M. (1976) Changes in differentiation of the urinary bladder during benign and neoplastic hyperplasia. In *Progress in Differentiation Research.* Ed. Muller-Berat *et al.* Amsterdam: North Holland. p. 339.

HICKS, R. M. (1977) Discussion of morphological markers of early neoplastic change in the urinary bladder. *Cancer Res.*, **37**, 2822.

HICKS, R. M. (1980) Multistage carcinogenesis in the urinary bladder. *Br. Med. Bull.*, **36**, 39.

HICKS, R. M., GOUGH, T. A. & WALTERS, C. L. (1978) Demonstration of the presence of nitrosamines in human urine: Preliminary observations on a possible aetiology for bladder cancer. In *Environmental Aspects of N-nitroso compounds.* Ed. Walker *et al.* Lyon: I.A.R.C. Publ. 19. p. 465.

HICKS, R. M., KETTERER, B. & WARREN, R. C. (1974) The ultrastructure and chemistry of the luminal plasma membrane of the mammalian urinary bladder. A structure with low permeability to water and ions. *Philos. Trans. R. Soc. Lond.* (*Biol.*), **268**, 23.

HICKS, R. M. & WAKEFIELD, J. ST J. (1976) Membrane changes during urothelial hyperplasia and neoplasia. *Cancer Res.*, **36**, 2502.

HICKS, R. M., WALTERS, C. L., ELSEBAI, I., EL-AASSER, A. B., EL-MERZABANI, M. & GOUGH, T. (1977) Demonstration of N-nitrosamines in human urine. *Proc. R. Soc. Med.*, **70**, 413.

HIGGINSON, J. & OETTLE, A. G. (1962) Cancer of the bladder in the South African Bantu. *Acta Un. Int. Contra. Cancrum*, **18**, 579.

HILL, M. J. & HAWKSWORTH, G. (1972) Bacterial production of nitrosamines *in vitro* and *in vivo*. In *N-Nitroso Compounds Analysis and Formation.* Ed. Bogosvki *et al.* Lyon: IARC Sci. Publ. **3**. p. 116.

HIROSE, M., FUKUSHIMA, S., HANANOUCHI, M. & 4 others (1976) Different susceptibilities of the urinary bladder epithelium of animal species to 3 nitroso compounds. *Gann*, **67**, 175.

HODGES, G. M., HICKS, R. M. & SPACEY, G. D. (1976) Scanning electron microscopy of cell-surface changes in methylnitrosourea (MNU)-treated rat bladders *in vivo* and *in vitro*. *Differentiation*, **6**, 143.

HOUSTON, W. (1964) Carcinoma of the bladder in Southern Rhodesia. *Br. J. Urol.*, **36**, 71.

ITO, N., HIASA, Y., TAMAI, A., OKAJIMA, E. & KITAMURA, H. (1969) Histogenesis of urinary bladder tumours induced by N-butyl-N-(4-hydroxybutyl)nitrosamine. *Gann*, **60**, 401.

JACOBS, J. B., ARAI, M., COHEN, S. M. & FRIEDELL, G. H. (1976) Early lesions in experimental cancer: Scanning electron microscopy of cell surface markers. *Cancer Res.*, **36**, 2512.

JORDON, P., VON LICHTENBERG, F. & GOATLY, K. D. (1967) Experimental schistosomiasis in primates in Tanzania: Preliminary observations on the susceptibility of the baboon *Papio anubis* to *Schistosoma haematobium* and *Schistosoma mansoni*. *Bull. WHO*, **37**, 393.

KUNTZ, R. E., CHEEVER, A. W. & MYERS, B. J. (1972) Proliferative lesions of the urinary bladder of non-human primates infected with *Schistosoma haematobium*. *J. Natl Cancer Inst.*, **48**, 223.

KUNTZ, R. E., CHEEVER, A. W., BRYAN, G. T., MOORE, J. A. & HUANG, T. (1978) Natural history of papillary lesions of the urinary bladder in schistosomiasis. *Cancer Res.*, **38**, 3836.

KUNTZ, R. E., MYERS, B. J., HUANG, T. C. & MOORE, J. A. (1971) Use of non-human primates in experimental schistosomiasis haematobium. *Proc. 3rd Int. Congr. Primatol.*, **2**, 162. Basle: Karger.

LAUGHLIN, L. W., FARID, Z., MANSOUR, N., EDMAN, D. C. & HIGASHI, G. I. (1978) Bacteriuria in urinary schistosomiasis in Egypt. A prevalence survey. *Ann. J. Trop. Med. Hyg.*, **27**, 916.

MILLIER, J. K. (1976) Growth rates of newly-imported baboons (*Papio anubis*). *Primate Supply* (*House Journal of Shamrock Farms Ltd*), **1**, 4.

NEWMAN, J. & HICKS, R. M. (1977) Detection of neoplastic and preneoplastic urothelia by combined scanning and transmission electron microscopy of urinary surface of human and rat bladders. *Histopathology*, **1**, 125.

PRATES, M. D. & TORRES, F. O. (1965) A cancer survey in Lourenco Marques, Portuguese East Africa. *J. Natl Cancer Inst.*, **35**, 729.

PREUSSMANN, R., NEURATH, G., WULF-LORENTZEN, G., DAIBER, D. & HENGY, H. (1964) Anferbemethoden und Dunnschicht-Chromatographie von organichen N-Nitrosoverbindungen. *Z. Anal. Chem.*, **202**, 187.

SADUN, E. H., VON LICHTENBERG, F. & BRUCE, J. I. (1966) Susceptibility and comparative pathology of ten species of primates exposed to infection with *Schistosoma mansoni*. *Am. J. Trop. Med. Hyg.*, **15**, 705.

SADUN, E. H., VON LICHTENBERG, F., CHEEVER, A. W., ERICKSON, D. G. & HICKMAN, R. L. (1970) Experimental infection with *Schistosoma haematobium* in chimpanzees: Parasitologic, clinical, serologic and pathologic observations. *Am. J. Trop. Med. Hyg.*, **19**, 427.

SMITHERS, S. R. & TERRY, R. J. (1965) The infection of laboratory hosts with cercariae of *Schistosoma mansoni* and the recovery of the adult worms. *Parasitology*, **55**, 695.

Thompson, P. E., Meisenhelder, J. E. & Najarian, H. (1962) Laboratory studies on the effects of tris (p-aminophenyl)carbonium salts, tris (p-aminophenyl)-methanol and lucanthone hydrochloride against *Schistosoma mansoni*. *Am. J. Trop. Med. Hyg.*, **11**, 31.

Vogel, H. (1967) Experimentelle Infektionen mit *Schistosoma haematobium* an Mangaben und Schipansen. *Ann. Soc. Belge. Med. Trop. Parasit. Mycol.*, **47**, 107.

Webbe, G. & James, C. (1971) The importation and maintenance of schistosomes of human and veterinary importance. *9th Symp. Br. Toxicol. Soc. Parasitol.* Oxford: Blackwell. p. 77.

Webbe, G., James, C. & Nelson, G. S. (1974) *Schistosoma haematobium* in the baboon (*Papio anubis*). *Ann. Trop. Med. Hyg.*, **68**, 187.

Webbe, G., James, C., Nelson, G. S., Smithers, S. R. & Terry, R. J. (1976) Acquired resistance to *Schistosoma haematobium* in the baboon (*Papio anubis*) after cercarial exposure and adult worm transplantation. *Ann. Trop. Med. Parasitol.*, **70**, 411.